# Relative social status alters the synchrony of attribute integration in altruistic decisions

**DOI:** 10.1016/j.isci.2025.111911

**Published:** 2025-01-27

**Authors:** Yinmei Ni, Jian Li

**Affiliations:** 1 School of Psychological and Cognitive Sciences and Beijing Key Laboratory of Behavior and Mental Health, Peking University, Beijing 100871, China; 2 IDG/McGovern Institute for Brain Research, Peking University, Beijing 100871, China

**Keywords:** Computer science, Social sciences, Psychology

## Abstract

Social status, which represents the relative dominance structure in societies, forms the backdrop against which most social decisions are made. Effective social decision-making demands flexible integration of decision attribute weight (importance of an attribute) and attribute latency (when attributes start to affect decisions). However, current understanding of how attribute weight and latency are influenced by relative social status is limited. In three experiments, we dynamically manipulated subjects’ relative social status before they engaged in an altruistic decision task and found that their altruistic behavior was better explained by a time-varying drift diffusion model, in which relative social status selectively modulated attribute latency but not attribute weights. Furthermore, prosocial subjects exhibited higher sensitivity to attribute latency in response to changes in relative social status compared with individualistic subjects. Our results introduce a new dimension to the computational mechanisms underlying the intricate interplay between relative social status and attribute integration.

## Highlights

• Attribute weight and latency (RST) can both affect altruistic decisions
• Relative social status selectively modulated attribute latency but not attribute weights
• Prosocial and individualistic subjects differ in how relative social status alters RST
• RST mediates correlation between social status and altruistic behavior in prosocial subjects

## Graphical abstract

**Figure:**
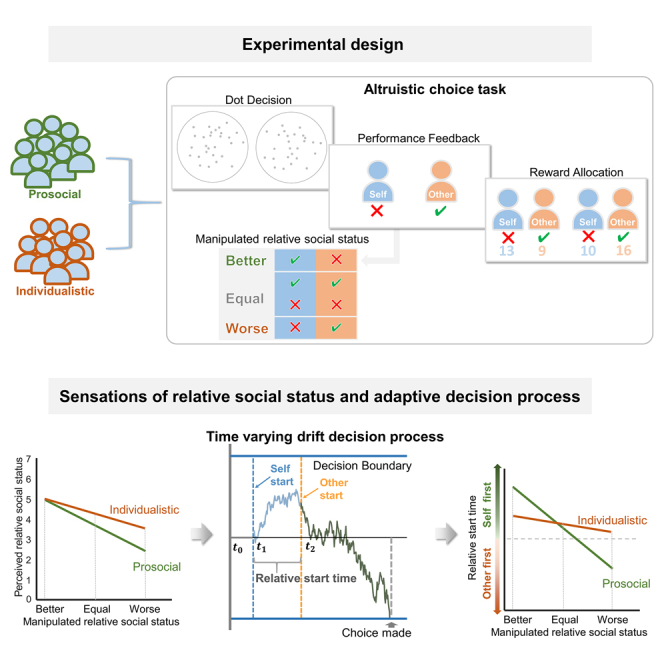

## Introduction

Social decisions are rarely made in a vacuum. In fact, most social decisions take place among individuals of certain social status, the hierarchical dominance structure in the societies.^1^ Adam Smith noted that people were willing to expend effort to attain certain social status, which was thought to be associated with the expectation of entitlement to resources.^2^ Indeed, social status affects the allocation of resources. For instance, sellers or buyers with endowed higher social status in an artificial market reap a greater share of the market surplus than their lower-rank counterparts.^1^ A key feature of social status is its relativity: employees in a corporation can both be supervisors to some colleagues and subordinates to others. Consequently, social decisions may be shaped by the dynamic and relative social status comparison.

In laboratory studies, relative social status is commonly induced via competitive performance or different endowments.^3^^,^^4^^,^^5^^,^^6^ One study manipulated relative social status by assigning different initial rewards to participants and found that, despite an aversion to social inequality, people accept more unequal offers in a money allocation choice game to avoid reversing their initial relative status.^5^ Other studies varied subjects’ social status by altering their performance relative to others in cognitive tasks. In these studies, subjects may first engage in dot number judgment or mathematical calculation tasks alongside other subjects, and social status is induced based on their relative task performance.^3^^,^^4^^,^^6^ These studies have shown that subjects’ social behavior can be adaptively adjusted in different status contexts. For instance, subjects were more likely to reject unfair allocations with higher relative social status compared to lower ones.^6^ Nevertheless, the cognitive and computational mechanisms underlying the impact of relative social status on social preferences, the degree to which other’s welfare is also taken into account when making a decision that has consequences to the payoffs of oneself and other people, remain poorly understood.

According to formal decision theories, social decision-making involves the integration of multiple attributes in the decision process.^7^^,^^8^^,^^9^^,^^10^^,^^11^^,^^12^^,^^13^^,^^14^^,^^15^^,^^16^^,^^17^^,^^18^ A well-established fact is that individuals care about not only their own welfare but also the welfare of others’ when faced with resource allocation decisions. A variety of utility models have been proposed by emphasizing different factors that influence the other-regarding behavior, including self-interest and inequity,^10^ selfishness and altruism,^7^^,^^15^ or equity and efficiency.^9^^,^^19^ These models assume that different attributes, combined with their subjective weights, constitute the speed of a noisy evidence accumulation process termed drift diffusion model (DDM), upon which decisions are triggered and response time (RT) patterns are yielded.^8^^,^^12^ However, such models, in which the relative impact of each attribute remains constant during the decision process, are agnostic to the empirical findings that subjects’ social preference may change as a function of RT.^20^^,^^21^^,^^22^

To address this question, dual-process models hypothesize that decisions evolve through a continuous arbitration between fast, automatic dispositional preferences and slower, reflective, and deliberate preferences during the decision process.^20^^,^^22^^,^^23^ Nonetheless, both correlational and causal evidence suggest that the preference for more prosocial choices is not monotonically associated with RT,^24^^,^^25^^,^^26^ raising the question of whether the deployment of dynamic attention, rather than automatic or deliberate preferences, influences choice RT in social dilemmas.^25^^,^^26^

Indeed, results from eye-tracking and computer mouse-tracking studies demonstrated that dynamic visual attention shaped how strongly certain options or attributes (such as tastiness or healthiness) influence food preference,^27^^,^^28^ and the revelation of food calorie information expedited the integration of healthiness attribute in the overweight individuals in a food-choice task.^29^ One study also incorporated eye-gaze information to the DDM in social decision-making tasks, highlighting the importance of attention in value integration and preference generation in altruistic decisions.^16^ Furthermore, recent studies using the multi-attribute, time-dependent DDM found that the temporal order in which different attributes are integrated during decision also affects choice and RT in both non-social^30^^,^^31^ and social decisions.^32^ Thus, it is likely that both the attribute weight (how important an attribute is to the decision-maker) and attribute latency (when an attribute starts to affect the decision process) are susceptible to the manipulation of social status and consequently to shape social preference across individuals and decision contexts. Delineating the channels via which relative social status exerts its influence in resource allocation tasks will pave the way to better understand how social preference dynamically adapts to different social contexts.

Here, we adopted a specific variant of DDM to formally investigate the computational mechanisms of how the relative social status affects subjects’ social preferences. Compared to the standard DDM, our model additionally introduced a parameter to capture the time difference between attribute latencies, the relative start time (RST).^28^^,^^30^^,^^31^^,^^32^^,^^33^ Therefore, this model (RST DDM) allows us to ascribe subjects’ choice and RT data to two separate psychological aspects in altruistic decisions: RST, how quickly one decision attribute starts to influence evidence accumulation in the decision process, and attribute weight, the relative importance of specific attributes influencing choice selection. Under this framework, flexible social decisions can be shaped by relative social status manipulation via changes in attribute weights, RST, or both.

**Figure 1:**
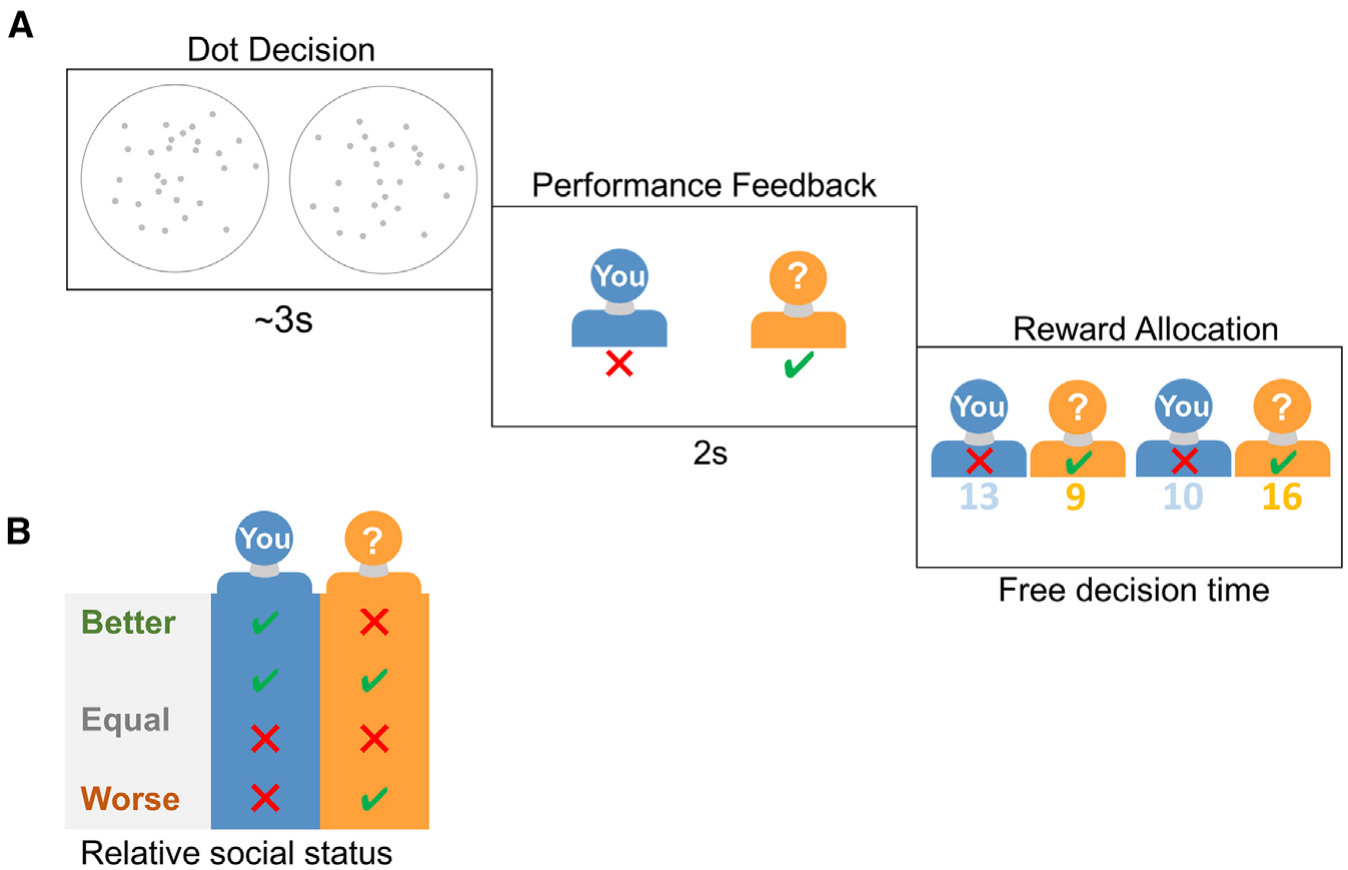
Task design (A) Altruistic choice task. In each trial, subjects first performed a dot number estimation task. Upon receiving feedback, they had to choose between two reward allocation options, each option engendering different payoffs for the subject and the co-player. (B) We manipulated subject’s relative social status by the relative dot performance between subjects and their co-players. Specifically, three relative social statuses (better, equal, and worse) were defined based on whether subjects’ performance was better than, equal to, or worse than their co-players’.

To test how the RST and attribute weights are affected by the relative social status, we designed a two-stage decision experiment that included a dot number estimation task to experimentally elicit different relative social statuses for the subjects and an altruistic choice task where subjects had to arbitrate between options entailing different monetary payoffs to themselves and co-players (Figure 1). We found that subjects made more altruistic choices under low relative social status compared to high status. The model-based analyses (RST DDM) reveal that the latency and weights of the two decision attributes, namely the payoffs to the self ($M_{s}$) and the other co-player ($M_{o})$, play dissociable and significant roles in the altruistic choice task. Interestingly, lower relative social status leads to earlier consideration of other’s payoff ($M_{o})$ relative to that of one’s own ($M_{s})$ but does not influence how strongly subjects evaluate their own or others’ payoffs. Furthermore, we show that the sensitivity of RST in response to the change of relative social status differed between prosocial and individualistic subjects who were classified based on their social value orientation (SVO) scale.^34^ The sensitivity is mainly driven by the more prosocial subjects in our experiment. The more individualistic subjects, however, have significantly higher weights toward their own payoff than the prosocials, but their RSTs are less sensitive to the change of relative social status than the prosocials. Finally, these results were replicated in two separate cohorts of subjects, confirming our hypothesis that relative social status affects social decisions via the modulation of attribute latencies (RST) as opposed to attribute weights.

## Results

### Relative social status modulates social preference

In our study, each trial subjects were paired with an anonymous co-player. After completing the dot number estimation task, subjects were asked to choose between two options entailing different $M_{s}$ and $M_{o}$ (Figure 1A). Based on the specific feedback from the dot estimation task (Figure 1B), we grouped trials into three relative social status categories (better, equal, and worse, see STAR Methods and Figure S1 for details) according to subjects’ relative performance to their co-players (Figure 1B).

**Figure 2:**
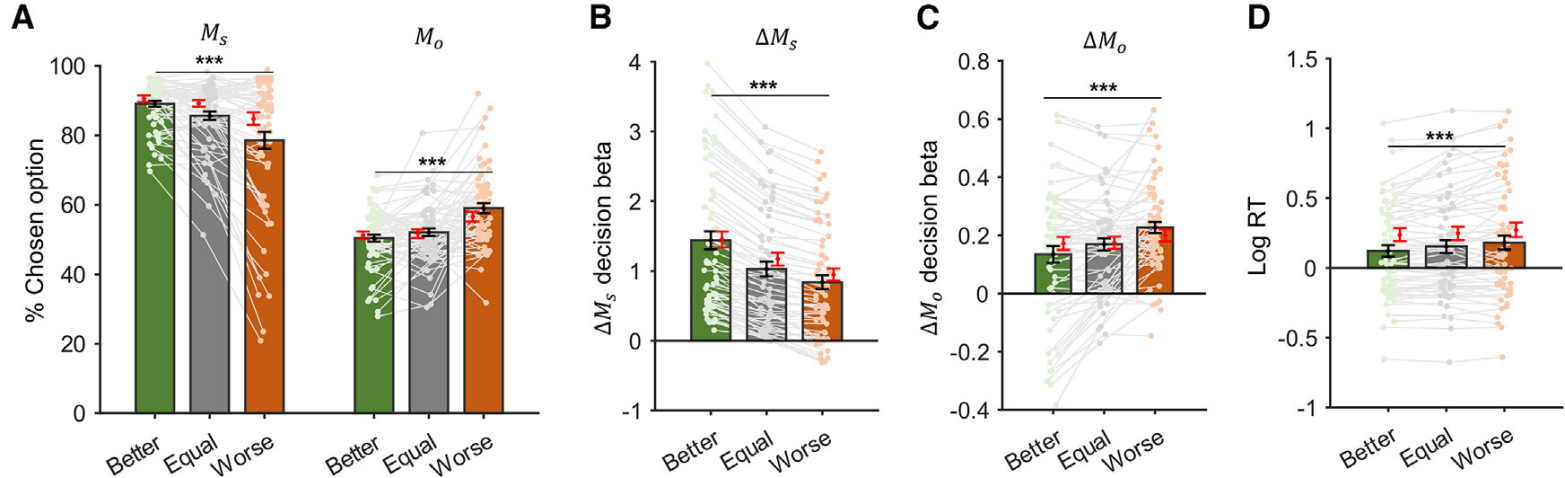
Behavioral results (A) Trial percentage of choosing larger self-payoff ($M_{s}$) or larger co-player’s payoff ($M_{o}$) options decreased and increased, respectively, as the relative social status worsened. (B and C) Decision betas of $\Delta M_{s}$ (B) and $\Delta M_{o}$ (C) for choices across three relative social status, where $\Delta M_{s}$ represents the $M_{s}$ difference between the lager and smaller $M_{s}$ options and $\Delta M_{o}$ represents $M_{o}$ difference between the lager and smaller $M_{s}$ options. (D) Mean log response time (RT). Black error bars represent SEM across subjects, and red error bars represent model predictions from the cross-validation results (see STAR Methods). ∗∗∗*p* < 0.001.

First, we examined how subjects’ relative social status influenced their prosocial behavior and found that subjects’ self-payoff ($M_{s}$) maximization behavior declined as their relative social status worsened. In the meantime, subjects were more likely to choose options that maximized their co-players’ payoffs ($M_{o}$, Figure 2A). One-way repeated ANOVA confirmed that the main effect of relative social status was significant for both choice types ($M_{s}$ maximization $:F\left(2,128\right)=23.167,P<0.001,\eta^{2}=0.266$; $M_{o}$ maximization: $F\left(2,128\right)=20.649,P<0.001,\eta^{2}=0.244$). Next, we conducted a mixed-effect logistic regression analysis to quantitatively examine the effect of relative social status on subjects’ social preferences by modeling their choices as a function of the difference of $M_{s}$ ($\Delta M_{s})$ and $M_{o}$ ($\Delta M_{o})$ between alternative options with larger and smaller $M_{s}$ (see STAR Methods for details). Our results showed that the decision betas (regression coefficients) of $\Delta M_{s}$ and $\Delta M_{o}$ were both positive and significant across different levels of relative social status (all p < 0.001), indicating that subjects’ choices were sensitive to both $\Delta M_{s}$ and ${\Delta M}_{o}$. Consistent with subjects’ choice behavior (Figure 2A), the relative social status exerted opposite effects on $\Delta M_{s}$ and $\Delta M_{o}\text{:}$ one-way ANOVA showed that the influence of $\Delta M_{s}$ declined with worsened relative social status ($\Delta M_{s}:F\left(2,128\right)=215.045,p<0.001,\eta^{2}=0.771$, Figure 2B), with the simultaneously increasing effect of $\Delta M_{o}$ ($\Delta M_{o}:F\left(2,128\right)=15.735,p<0.001{,\eta }^{2}=0.197$, Figure 2C), suggesting that subjects became more prosocial as their relative social status decreased. Finally, the mean RT increased significantly as relative social status worsened (Figure 2D, log RT :F(2,128)=10.277,p<0.001*,* $\eta^{2}=0.161$). These results on the surface support the hypothesis that relative social status modulates decision betas on different decision attributes ($\Delta M_{s}$ and $\Delta M_{o}$). Given the larger magnitude of the decision beta on $\Delta M_{s}$ relative to that of $\Delta M_{o}$ (Figures 2B and 2C), worsening relative social status, according to the standard DDM, would lead to more commensurate attribute weights of $\Delta M_{s}$ and $\Delta M_{o}$ and therefore prolong the decision process with longer RT. However, recent development in evidence accumulation models suggests that the rate of evidence accumulation (attribute weights) and the time attributes entering the decision process can have dissociable impacts on subjects’ choices and RT.^30^^,^^31^^,^^33^

### Time-varying drift diffusion process modeling

**Figure 3:**
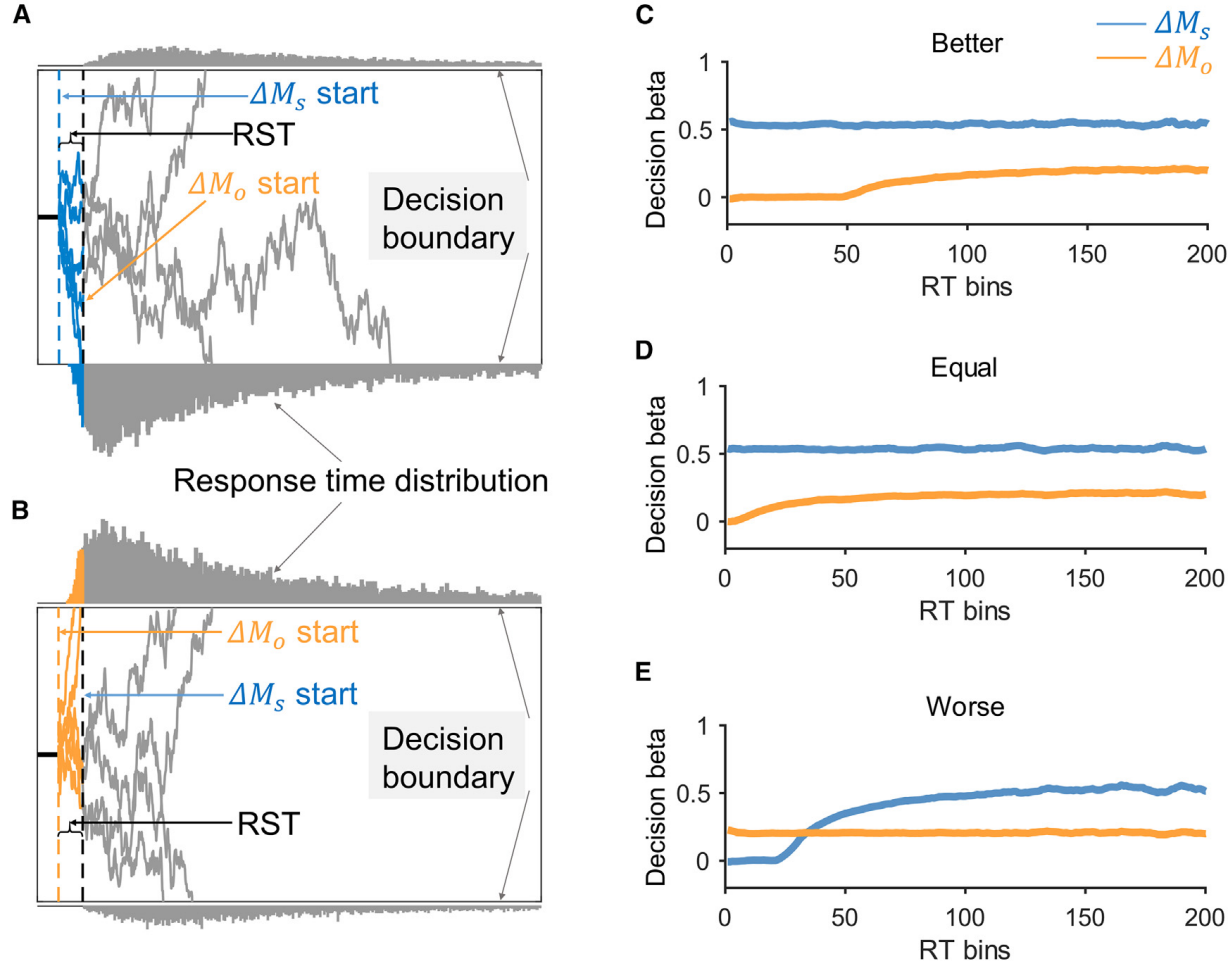
Model illustration and prediction (A and B) are graphic illustrations of the relative start time drift diffusion model (RST DDM). (A) Illustration of when $\Delta M_{s}$ enters the decision process first followed by $\Delta M_{o}$, and their start time difference is the relative start time (RST). A decision is made when the accumulated evidence reaches the decision boundary. Here we show that two agents with the same drift weight for the two attributes but different attribute latency ($\Delta M_{s}$ earlier in A and $\Delta M_{o}$ earlier in B) could result in different choices. Blue, evidence accumulation trajectory where only $\Delta M_{s}$ is considered in the decision process; orange, only $\Delta M_{o}$ is considered; gray, both attributes accumulate in the decision process. The gray distributions represent RT histograms for different types of choices. (C–E) are RST DDM simulated attribute effects (decision betas) as a function of response time (RT) for both $\Delta M_{s}$ (blue) and $\Delta M_{o}$ (yellow) in better, equal, and worse social conditions. The x axis represents overlapping 100 ms RT windows that slide along RT in steps of 10 ms.

To systematically investigate how relative social status affects subjects’ social preferences, we adopted a sequential sampling model in which both the weight and latency of decision attributes ($\Delta M_{s}$ and $\Delta M_{o}$) could be influenced by the relative social status. The latency or the time of different attributes entering the decision process effectively controls the temporal dynamics of attribute weighting strength during choice. Similar to earlier research developing such a framework,^35^^,^^36^^,^^37^^,^^38^^,^^39^^,^^40^^,^^41^ we extended the classic DDM by introducing an additional free parameter describing how fast decision attribute $\Delta M_{s}$, relative to $\Delta M_{o}$, begins to be considered in the evidence accumulation process^30^^,^^31^^,^^32^^,^^33^ (RST, see STAR Methods for details). Under this framework, the RST parameter, in combination with attribute weight, determines subjects’ social preference and RT distribution (RST DDM). Figure 3A illustrates an example where $\Delta M_{s}$ enters the decision process earlier than $\Delta M_{o}$. According to the RST DDM, choices made before $\Delta M_{o}$ is considered are predominantly influenced by the evidence accumulation rate of $\Delta M_{s}$ (blue traces). On the contrary, $\Delta M_{o}$ dominates choice selection before $\Delta M_{s}$ enters the decision process (Figure 3B, orange traces). Such effects are further reflected on the dynamic decision betas (logistic regression coefficients) of both $\Delta M_{s}$ and $\Delta M_{o}$ on subjects’ choice behavior as a function of RT (Figures 3C–3E). More specifically, time-varying decision betas are observed when $\Delta M_{s}$ starts influencing the decision process earlier (Figure 3C, orange trace $\Delta M_{o}$) or later (Figure 3E, blue trace $\Delta M_{s}$) than $\Delta M_{o}$. Such variability is caused by the asynchrony of $\Delta M_{s}$ and $\Delta M_{o}$ in terms of their RSTs in influencing the evidence accumulation process. Indeed, simultaneous consideration of both $\Delta M_{s}$ and $\Delta M_{o}$ yields relatively stable decision betas for different attributes ($\Delta M_{s}$ and $\Delta M_{o}$) during the decision process (Figure 3D).

Recently, this approach has been applied to studies of individual preference formation involving the trade-off between attributes such as tastiness and healthiness when contemplating food options,^30^ and contact avoidance and monetary compensation following interpersonal transgression.^42^ We adopted this framework to investigate whether and, if so, how the relative social standing would influence RST in the decision process and subsequently determine subjects’ social preference. Based on the behavioral effects in Figure 2, three hypotheses related to the influence of relative social status arise: (1) relative social status modulates both the evidence accumulation rate (drift weight) and the RST of different attributes ($\Delta M_{s}$ and $\Delta M_{o}$), (2) it only modulates the attribute RST but not the drift weight, and (3) it specifically modulates drift weight but has no effect on RST. To test these different possibilities, we built a series of RST DDMs by including a RST(RST: the start time difference between $\Delta M_{s}$ and $\Delta M_{o}$) parameter in the standard DDM. In model 1, we assume that both $\Delta M_{s}$ and $\Delta M_{o}$ enter the decision process simultaneously (RST = 0) and relative social status modulates the individual drift weights of $\Delta M_{s}$ and $\Delta M_{o}$ (model 1, see STAR Methods, Equation 1). Furthermore, RST might be different across relative social status and $\Delta M_{s}$ and $\Delta M_{o}$ share the same drift weight (model 2). Finally, it is also possible that relative social status modulates the individual drift weights of $\Delta M_{s}$ and $\Delta M_{o}$ separately but with a constant RST (model 3) or status-related RSTs (model 4). All the aforementioned models, in principle, can produce qualitatively similar behavioral patterns such as status-dependent reaction time and decision betas (Figure 2).

To quantitatively examine the resolution of the decision process, we used the differential evolution algorithm^43^ (see STAR Methods for details) to interrogate the candidate models against both behavioral choices and RTs of subjects’ data. This algorithm has been applied previously in both social and non-social preference tasks and can distinguish the latency and weight of different attributes influencing choices.^31^^,^^44^ We compared our candidate models by performing a Bayesian model comparison^45^ test, and the model with social status-dependent RSTs but status-independent drift weights outperformed all other models (model 2: exceedance probability [EXP] = 0.999, Figure S2A). Further parameter recovery analysis showed that all the model parameters recovered well from simulated choice dataset with sufficient true parameter ranges of our winning model (RST model; Figure S3).

We then conducted a series of analyses to test whether the RST model (model 2) captured the characteristics of behavioral patterns of our subjects. First, we ran a cross-validation analysis to test the out-of-sample accuracy of our model in terms of predicting prosocial choices as well as RTs. We estimated the best-fitting parameters of the model from a randomly selected half of the trials (for each relative social status) for each subject and tested the accuracy of model predictions against the other half of the trials within each social status condition (see STAR Methods for details). Model predictions captured the overall RT patterns in different relative social status (Figure 2D, one-way repeated ANOVA, F(2,128)=8.823,p<0.00 *1,* $\eta^{2}=0.121$, red error bars) as well as the inter-subject difference within each relative social status (Figure S4). Furthermore, our model also captured subjects’ increasing prosocial preference as their relative social status decreased (Figure 2A, larger self-payoff ($M_{s})$ option proportion: $F\left(2,128\right)=14.183,p<0.001,\eta^{2}=0.181\text{;}$ larger co-player payoff ($M_{o})$ option proportion: $F\left(2,128\right)=12.258,p<0.001,\eta^{2}=0.161$, red error bars). The regression analysis also confirmed this pattern: simulated choice data from our winning RST model revealed status-dependent decision weights on both $\Delta M_{s}$ (Figure 2B, $F\left(2,128\right)=205.167,p<0.001,\eta^{2}=0.762$, red error bars) and $\Delta M_{o}$ (Figure 2C, F(2,128)=6.555,p=0.002*,* $\eta^{2}=0.093$, red error bars), suggesting that the asynchrony of different decision attributes (RST) can sufficiently account for the choice and RT patterns we observed across relative social status.

### Model parameters and inter-subject behavioral variance

**Figure 4:**
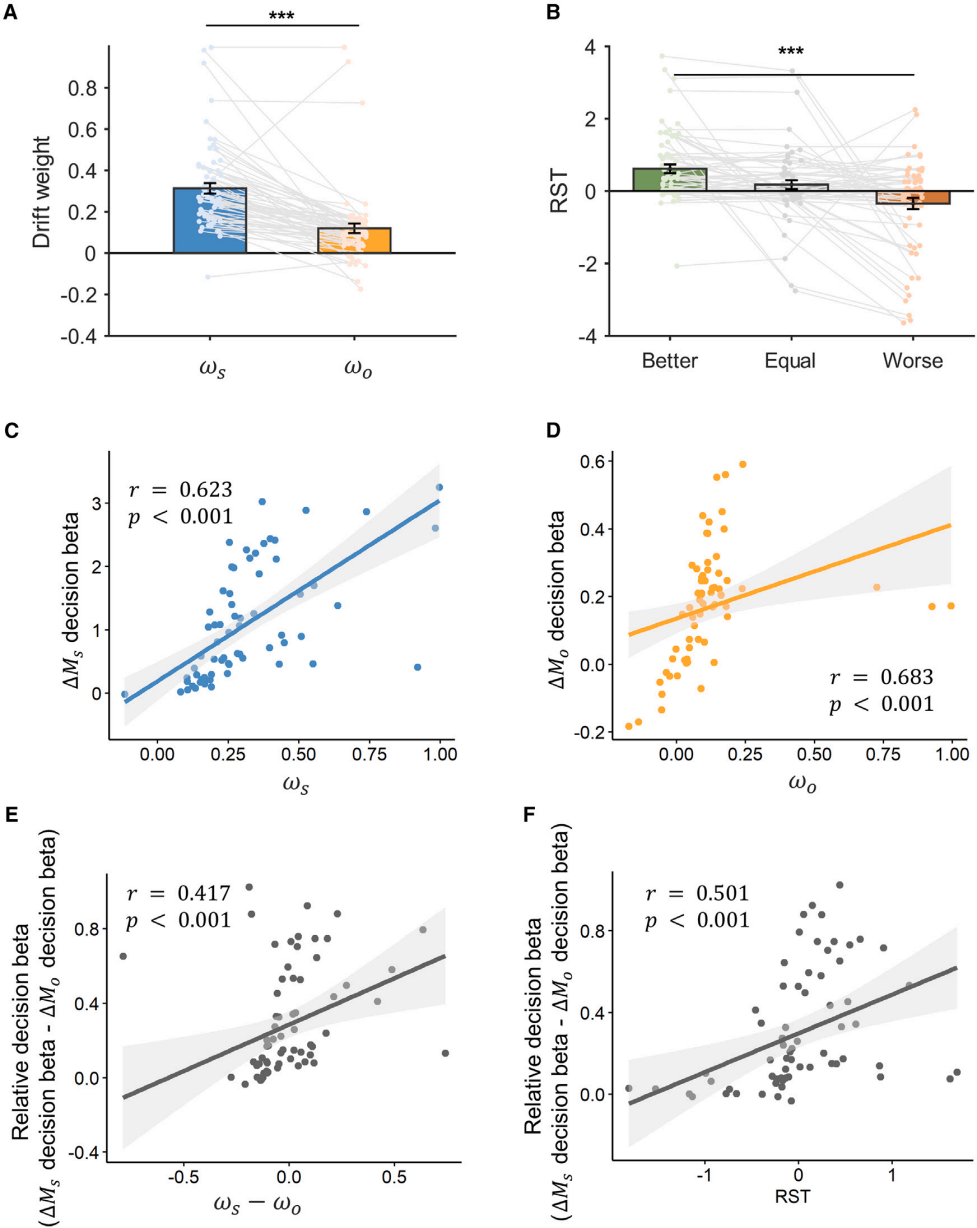
RST DDM parameters and correlation with decision betas (A) Model-estimated drift weight for $\Delta M_{s}$ ($\omega_{s}$) and $\Delta M_{o}\left(\omega_{o}\right)$. (B) Model-estimated RST, the relative start time between $\Delta M_{s}$ and $\Delta M_{o}$. Positive RST indicates earlier entry of $\Delta M_{s}$ in the decision process, whereas negative RST represents earlier entry of $\Delta M_{o}$. (C and D) Correlations between drift weights ($\omega_{s}$ and $\omega_{o}$) and decision betas for $\Delta M_{s}$ (C) and $\Delta M_{o}$ (D). (E) Correlation between the relative drift weight and relative decision beta, controlling for the effect of RST. (F) Correlation between RST and relative decision beta, controlling for the effect of relative drift weight. ∗∗∗*p* < 0.001, ∗*p* < 0.05. Error bars represent SEM across subjects.

Further examination of the best-fitting model (model 2) suggests that higher drift weights are assigned to the $\Delta M_{s}$ than $\Delta M_{o}$ and this asymmetry does not change across relative social status (Figure 4A, paired t test, $t_{64}=7.406,p<0.001,{\;cohen}^{\prime }s\;d=0.919$). On the other hand, the influence of relative social status is manifested as the differential RSTs: as the relative social status decreases, RST starts to decrease (Figure 4B, one-way repeated ANOVA, $F\left(2,128\right)=13.317,p<0.001,\eta^{2}=0.172$). Since RST is calculated as the difference between the start time of $\Delta M_{o}$ and $\Delta M_{s}$, the decrease of RST suggests that the lower relative social status facilitates earlier processing of other-regarding information ($\Delta M_{o})$. On the contrary, better relative social status expedites the processing of self-payoff ($\Delta M_{s}$).

Interestingly, this model captures not only social status-related behavioral differences but also inter-subject behavioral variance across different relative social status. Decision betas for ${\Delta M}_{s}$ and $\Delta M_{o}$, the model-free estimates of the influence of ${\Delta M}_{s}$ and $\Delta M_{o}$ on choice behavior, show strong correlation with model-estimated drift weights of ${\Delta M}_{s}$ (Figure 4C, robust correlation, r=0.623,p<0.001) and $\Delta M_{o}$ (Figure 4D, r=0.683,p<0.001), respectively, across relative social status as well as within each status (Figures S5A–S5F). Similar correlations are also observed when the relative decision betas between ${\Delta M}_{s}$ and $\Delta M_{o}$ are regressed against the relative drift weights of ${\Delta M}_{s}$ and $\Delta M_{o}$ (Figure 4E, robust correlation, r=0.417,p<0.001) or against RSTs (Figure 4F, r=0.501,p<0.001) both across relative social status and within each status condition (see Figures S5G–S5I for details). The aforementioned results suggest that status-dependent RSTs are responsible for both the social status-related behavioral variance (Figure 2) and the inter-subject behavioral difference across conditions (Figure 4F). Across subjects, those whose social attributes ($\Delta M_{o}$) enter earlier into the decision process show relatively higher behavioral influence by $\Delta M_{o}$ (Figure 4F). Additionally, there is a significant interaction effect between relative (${\Delta M}_{s}-\Delta M_{o}$) drift weights and RSTs on the relative decision betas (β=0.734,p=0.008), indicating that RST also exhibits a modulation effect of the drift weights on the decision betas, amplifying the influence of drift weights on subjects’ actual choice preference. We also tested the relationship between subjects’ interpersonal reactivity index (IRI) and the model parameters (drift weights for ${\Delta M}_{s}$ and $\Delta M_{o}$, RST slope across social status levels). Since IRI contains four subscales (perspective taking, fantasy, empathy concern, and personal distress), our results showed that none of the subscales was significantly correlated with the model parameters after multiple comparison correction (all p > 0.05, Bonferroni correction).

**Figure 5:**
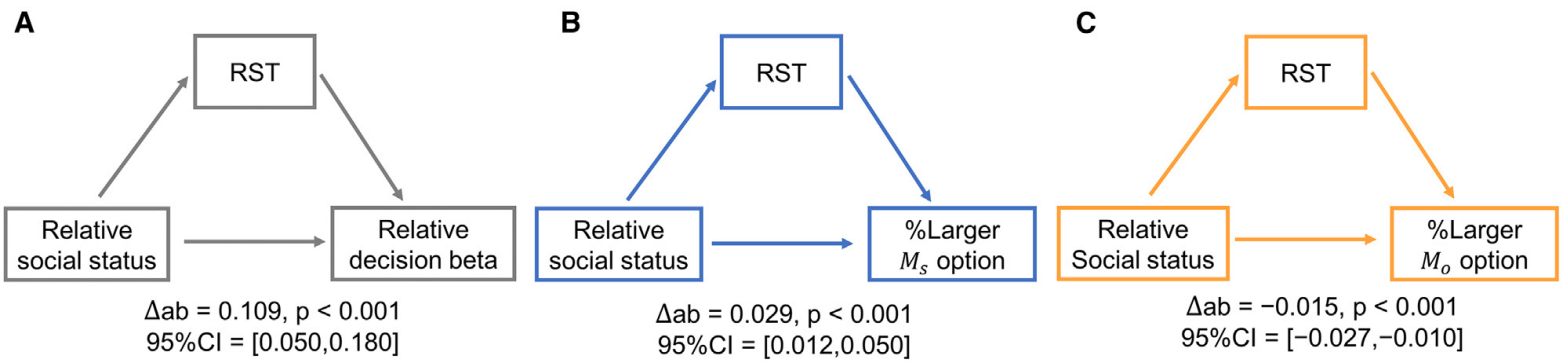
Mediation analysis (A) RST partially mediated the effect of relative social status on subjects’ relative decision beta ($\Delta M_{s}$ decision beta − $\Delta M_{o}$ decision beta). Similarly, RST partially mediated the effect of relative social status on subjects’ choosing larger $M_{s}$ options (B) or larger $M_{o}$ option (C). $\Delta M_{s}$ and $\Delta M_{o}$ represent the difference $M_{s}$ ($\Delta M_{s})$ and $M_{o}$ ($\Delta M_{o})$ between alternative options with larger and smaller $M_{s}$, respectively.

Given RSTs’ explanatory power in explaining relative decision betas within (Figures 2B and 2C) and across (Figure 4F) relative social status, we further hypothesize that RSTs might directly mediate the correlation between relative social status and subjects’ prosocial preferences. Therefore, we ran a mediation analysis, and it revealed that RSTs mediated the effect of relative social status on the relative (${\Delta M}_{s}-{\Delta M}_{o}$) decision betas (Figure 5A, Δab=0.109,p⟨0.001,95%CI=[0.050,0.180]) after controlling for the effects of drift weights. Similarly, RSTs also mediated the effect of relative social status on subjects’ prosocial preference measured by selecting either options with larger $M_{s}$ (Figure 5B, Δab=0.029,p⟨0.001,95%CI=[0.012,0.050]) or options with larger $M_{o}$ (Figure 5C, Δab=−0.015,p⟨0.001,95%CI=[−0.027,−0.010]), again controlling for the confounders of drift weights’ effects. These results further confirm the key role of RSTs in determining subjects’ prosocial preference.

### Interaction of social status and SVO on decision processes

Since relative social status has a direct effect on people’s prosocial behavior and the degree of SVO has been shown to be a reliable predictor of subjects’ prosocial behavior,^46^^,^^47^ we further examined whether the relative social status-related social preference was modulated by subjects’ SVO. To test this, we first categorized subjects into prosocial (*n* = 35) and individualistic (*n* = 30) groups based on their SVO angle^34^ (see STAR Methods) and then compared the choice and RT differences between the two groups. A model-free two-way ANOVA (SVO type (2) × relative social status (3)) of choice proportions revealed that prosocials were less likely to maximize $M_{s}$ (SVO main effect: $F\left(1,63\right)=8.643,p=0.005,\eta^{2}=0.121$, Figure S6A) and more likely to choose options with larger $M_{o}$ (Figure S6B, SVO main effect $F\left(1,63\right)=12.458,p=0.001,\eta^{2}=0.165$) than the individualistic subjects. Interestingly, the interaction effects of relative social status and SVO were also significant in subjects’ choice preferences (Figure S6A, $F\left(2,126\right)=3.691,p=0.028,\eta^{2}=0.055$; Figure S6B, $F\left(2,126\right)=10.999,p<0.001,\eta^{2}=0.149$), suggesting that relative social status might exert different effects on subjects’ social preference contingent on their prosociality. Similar main effects of SVO and the interaction of SVO and relative social status were also observed on the decision betas of ${\Delta M}_{s}$ (Figure S6C, SVO main effect: $F\left(1,63\right)=15.607,p<0.001,\eta^{2}=0.199$; interaction effect: $F\left(2,126\right)=12.028,p<0.001,\eta^{2}=0.160)$ and ${\Delta M}_{o}$ (Figure S6D, SVO main effect: F(1,63)=1.322,p=0.255*,* $\eta^{2}=0.021$; interaction effect: F(2,126)=5.836,p=0.004*,* $\eta^{2}=0.085$).

**Figure 6:**
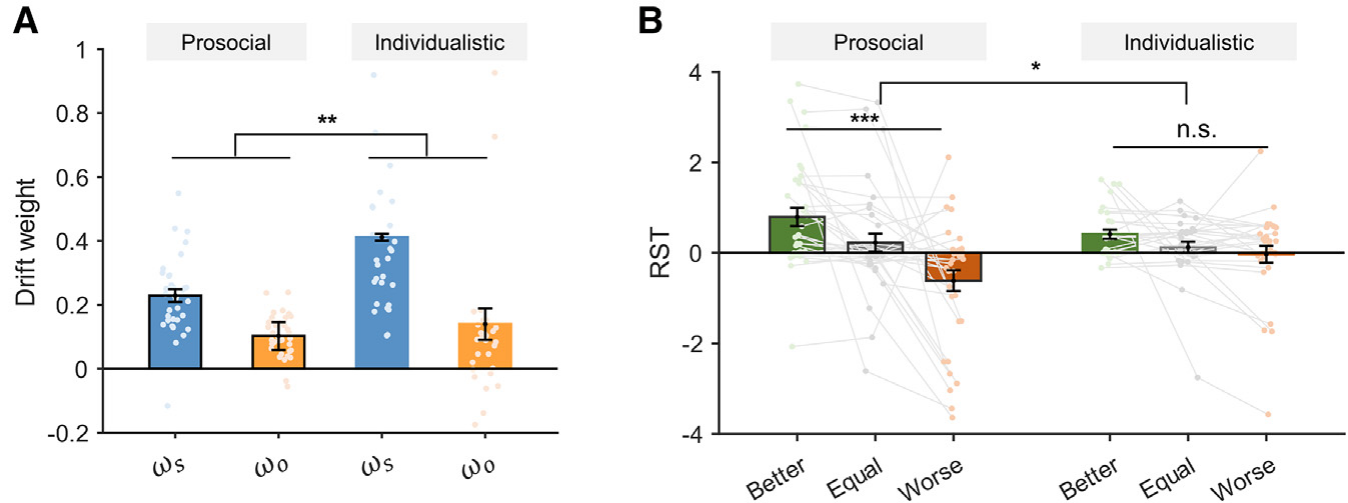
Model-estimated differences between prosocial and individualistic groups (A) Significant interaction indicates that the relative drift weights ($\omega_{s}-\omega_{o}$) in individualistic group are higher than those of the prosocial group. Asterisks across prosocial and individualistic groups denote significant interaction effect (attribute type × SVO group). (B) The relative start time (RST) between $\Delta M_{s}$ and $\Delta M_{o}$ attributes of the prosocial group was more sensitive to the changes in relative social status compared to that of the individualists. Asterisks across better, equal, and worse relative social status within each group denote simple effect analysis results. n.s., non-significant; ∗*p* < 0.05, ∗∗*p* < 0.01, and ∗∗∗*p* < 0.001. Error bars represent SEM across subjects.

Our model-based analysis further confirmed the earlier results. For both groups, we ran Bayesian model comparison separately, and the RST model (model 2) outperformed all the other candidate models in both groups (Figures S2B and S2C, prosocial group: EXP = 0.941; individualistic group: EXP = 0.999). Best-fitting model parameters in the RST model showed the general pattern of the drift weight difference between attributes ${\Delta M}_{s}$ and ${\Delta M}_{o}$ in both groups (Figure 6A, prosocial: $t_{34}=5.528$*,* p<0.002*,* cohen’sd=1.370; individualistic: $t_{29}=4.123$, p<0.002, cohen’sd=0.983, Bonferroni correction). However, the interaction effect of SVO (prosocial vs. individualistic) and attribute (${\Delta M}_{s}$ vs. ${\Delta M}_{o}$) types was also significant (F(1,63)=29.451,p<0.001, aligned rank test), indicating an even more biased attribute accumulation process in the individualistic group, which was also confirmed by the short RTs in this group (Figure S6E, $F\left(1,63\right)=16.215,P<0.001,\eta^{2}=0.205$). Lastly, we examined how relative social status and SVO influenced subjects’ RST in a two-way ANOVA. Although the SVO type did not yield a main effect on RST (Figure 6B, $F\left(1,63\right)=0.046,P=0.832,\eta^{2}=0.001$), the interaction of SVO and relative social status was significant ($F\left(2,128\right)=3.669,P=0.028,\eta^{2}=0.055$), suggesting that RST of the individualistic subjects was less affected by relative social status. Indeed, closer examination of each SVO group showed that the effect of relative social status was significant in prosocials ($F\left(2,68\right)=11.153,P<0.001,\eta^{2}=0.247$, Bonferroni correction) but did not reach statistical significance in the individualistic group ($F\left(2,58\right)=2.938,P=0.087,\eta^{2}=0.092$, Bonferroni correction). Taken together, these results demonstrate that not only the RST model captures the behavioral patterns of individual differences across subjects (Figure 2) but also its validity is reflected within both prosocial and individualistic groups. Similarly, our simulation results confirm that the winning RST model also captures the behavioral choice pattern and RT in both groups (Figure S6, red error bars).

### Confirmation of model validity in two replication studies

**Figure 7:**
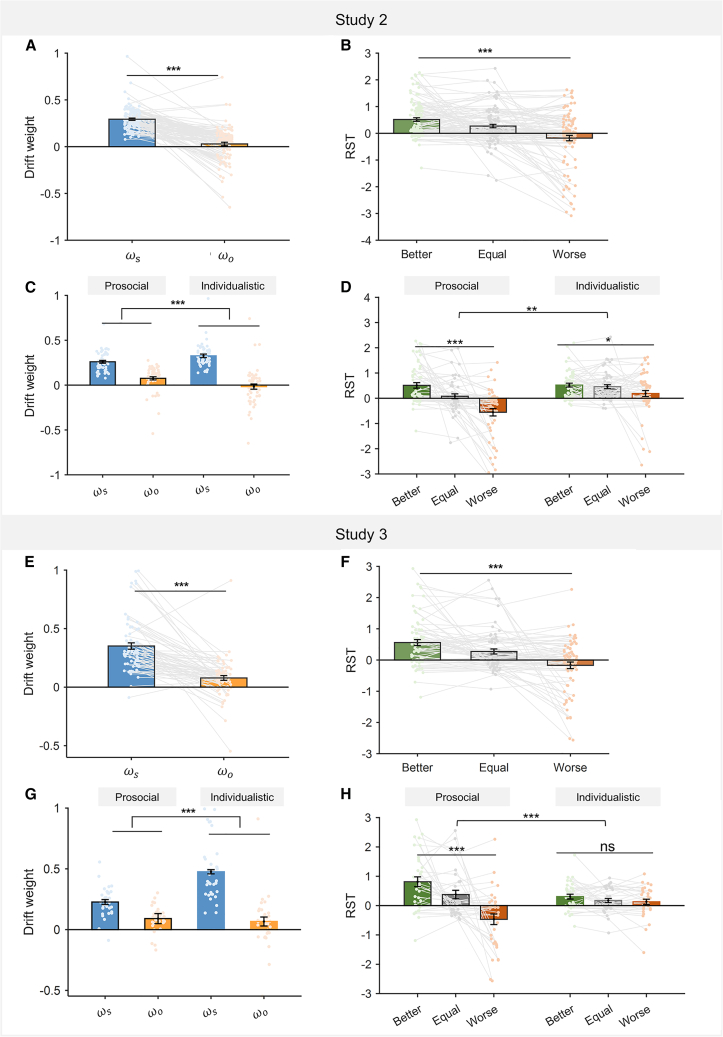
Modeling results for studies 2 and 3 (A and E) Model-estimated drift weight of $\omega_{s}$ and $\omega_{o}$ for $\Delta M_{s}$ and $\Delta M_{o}$ attributes, respectively. (B and F) Model-estimated relative start time (RST) between $\Delta M_{s}$ and $\Delta M_{o}$ attributes. (C and G) Prosocial and individualistic groups differ in their relative drift weights. Significant interaction indicates that the relative drift weights ($\omega_{s}-\omega_{o}$) of the individualistic subjects are higher than those of the prosocials. (D and H) The RST of the prosocial group was more sensitive to the changes in relative social status compared to that of the individualistic group. Asterisks across better, equal, and worse status within each group denote simple effect analysis results. n.s., non-significant; ∗*p* < 0.05, ∗∗*p* < 0.01, and ∗∗∗*p* < 0.001. Error bars represent SEM across subjects.

To further verify the validity of our results and the reliability of the RST model in accounting for the relative social status effects, we replicated the study with two additional datasets (study 2: *n* = 105, 52 prosocial and 53 individualistic subjects; study 3: *n* = 68, 34 prosocial and 34 individualistic subjects; see STAR Methods). In the two studies, we replicated all the major findings from the original study (Figures 7 and S7–S15; Tables S1 and S2): the relative social status modulated subjects’ social preference as well as their RT in both prosocial and individualistic groups (Figures S7 and S8), and the RST model (model 2) performed the best among the candidate models (Figure S9). Again, we found significantly larger drift weight of ${\Delta M}_{s}$ than ${\Delta M}_{o}$ attribute (study 2: $t_{104}=12.694,p<0.001,{cohen}^{\prime }s\;d=1.239\text{,}$ Figure 7A; study 3: $t_{67}=7.734,p<0.001,{cohen}^{\prime }s\;d=0.938$, Figure 7E), and RST decreased significantly as relative social status worsened (study 2: $F\left(2,208\right)=22.426,p<0.001,\eta^{2}=0.177$, Figure 7B; study 3: $F\left(2,134\right)=17.415,p<0.001,\eta^{2}=0.206$, Figure 7F). Furthermore, the two-way aligned rank test also revealed a more biased processing of ${\Delta M}_{s}$ than ${\Delta M}_{o}$ in individualistic subjects (study 2: interaction effect: F(1,103)=35.176,p<0.001, Figure 7C; study 3: interaction effect: F(1,66)=33.285,p<0.001, Figure 7G). Most importantly, there were significant interaction effects of relative social status and SVO type on the RST in both replication studies (study 2: $F\left(2,206\right)=6.308,p=0.002,\eta^{2}=0.058$, Figure 7D; study 3: $F\left(2,132\right)=12.071,p<0.001,\eta^{2}=0.155$, Figure 7H), confirming similar attribute latency patterns: compared to the individualistic subjects, prosocial subjects’ RSTs were more sensitive to changes in relative social status (Figures 7D and 7H).

### Perceived social status drives the mediation effect of RST

**Figure 8:**
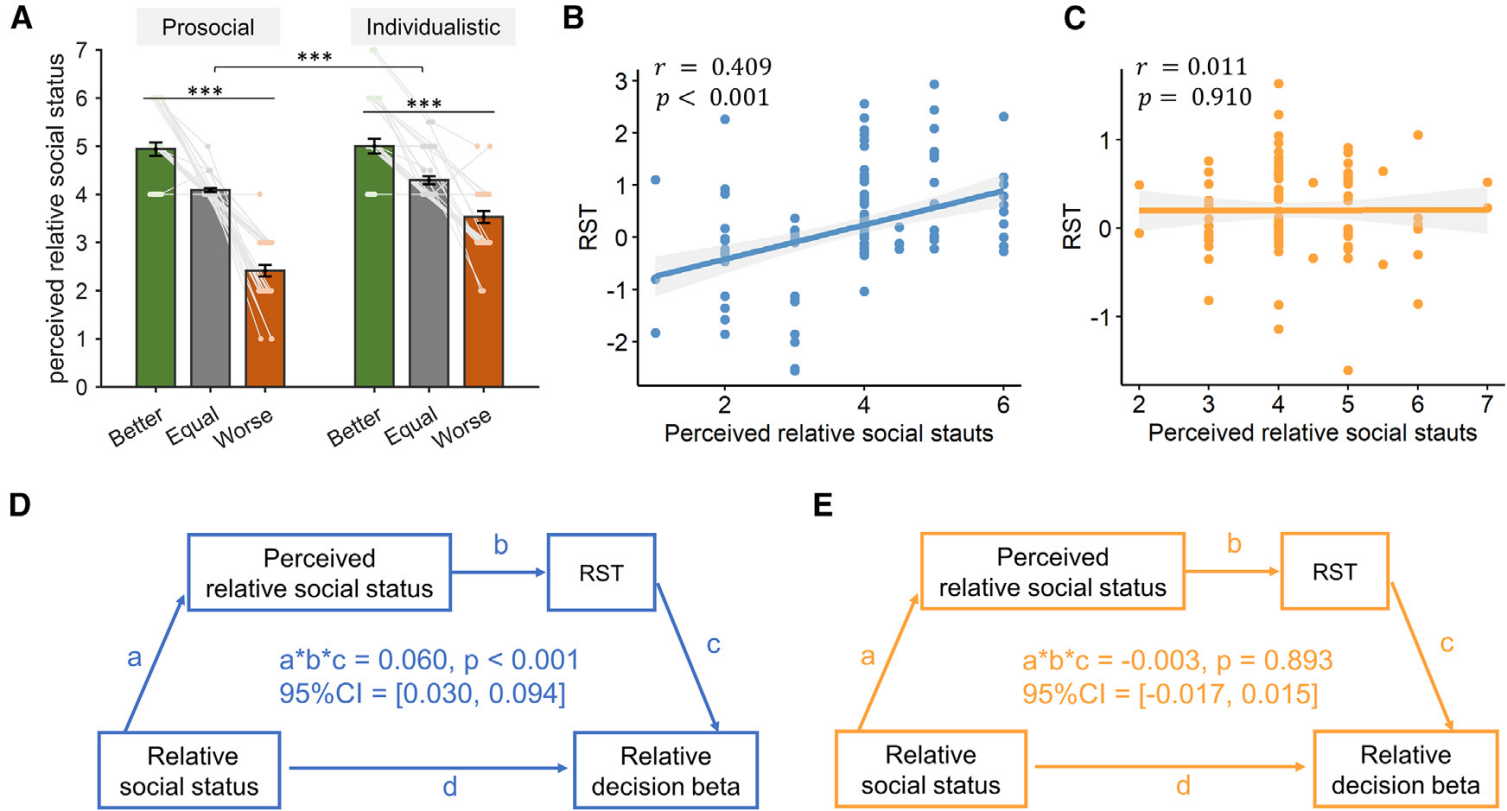
The perceived relative social status in study 3 (A) The perceived relative social status differed between prosocial and individualistic groups. (B and C) (B) The correlation between perceived relative social status and the relative start time (RST) was significant in the prosocial group, (C) but not in the individualistic group. ∗∗∗*p* < 0.001. Error bars represent SEM across subjects. (D and E) (D) The chain mediation analysis further revealed that the perceived relative social status and RST mediated the relative social status’s effect on relative decision beta (decision beta of $\Delta M_{s}$ − decision beta of $\Delta M_{o}$) in the prosocial group, (E) but not in the individualistic group.

In studies 1 and 2, participants were not asked about how they perceive their relative social status after receiving feedback from the dot estimation task. The higher RST sensitivity related to the change of social status in the prosocial group may be due to heightened subjective perception of the social status change in the prosocials relative to the individualistic subjects. To test such a possibility, we conducted study 3 where in addition to the dot estimation and altruistic choice tasks as in studies 1 and 2, subjects were also asked to report how they perceived their relative social status between them and their co-players on a seven-point scale (1 = very low, 7 = very high) for each type of feedback (better, equal, and worse) received in the dot estimation task. Two-way non-parametric aligned rank test^48^^,^^49^ (SVO type × social status) on subjects’ perceived social status showed a main effect of SVO (F(1,66)=29.025,p<0.001, Figure 8A), with individualistic subjects perceiving a higher relative social status than the prosocials. The main effect of social status manipulation was also significant (F(2,132)=255.692,p<0.001, Figure 8A), confirming the validity of the dot estimation task in eliciting different perceived social status. Furthermore, the interaction effect of SVO type × social status was also significant (F(2,132)=11.862,p<0.001), indicating that the prosocials were more sensitive to the changes of social status than the individualistic subjects (Figure 8A). Moreover, there was a significant correlation between perceived relative social status and RST in prosocials (robust correlation, r=0.409,p<0.002, Bonferroni correction, Figure 8B). However, this correlation was not significant in individualistic subjects (robust correlation, r=0.011,p=0.910, Figure 8C) and the interaction effect of SVO × perceived social status on RST was significant (interaction beta = 0.356, p<0.001), indicating that the higher RST sensitivities to the social status manipulation may be due to the higher perceived social status sensitivities in the prosocials. To test such a possibility, we ran a chain mediation model where the effect of social status manipulation on the relative decision beta (Figure 5A) was mediated via the path of perceived social status and RST. Confirming our hypothesis, such a mediation effect was indeed significant in the prosocial group (patha∗b∗c=0.060,p⟨0.001,95%CI=[0.030,0.094], Figure 8D) but not in the individualistic group (patha∗b∗c=−0.003,p=0.893,95%CI=[−0.017,0.015], Figure 8E). Similar difference between the two groups was also evident in subjects’ choices of larger self and other-payoff proportions (Figure S16).

## Discussion

Does the relative social status influence people’s social preference? Our results suggest that it does, but in a more nuanced manner. With the combination of relative social status manipulation and computational modeling, our findings provide empirical support to delineate two possible pathways through which the relative social status might influence social preference: when and how strongly the self (${\Delta M}_{s}$) and other-regarding (${\Delta M}_{o}$) attributes affect decisions. We have shown that the relative social status modulation was specifically associated with the RST of decision attributes entering the decision process: as relative social status worsened, the processing of other-regarding attribute was expedited compared to the self-attribute.

Previous literature on social status and morality has shown that higher social class is a good predictor for unethical behaviors including lying in negotiations, cheating, and law breaking.^50^^,^^51^ Subsequent studies further demonstrate that context-specific relative social status such as winning a competition also prompts subjects to behave more dishonestly,^52^ indicating that both stable and dynamic social status can predict moral choices. Conversely, people are averse to the transgression of the established relative social status via social welfare distribution.^5^ What remains unaddressed is how the social preference, usually considered as a stable trait,^34^ can adapt dynamically given the relative social status, upon which the social interaction occurs, is always context dependent.^6^ That is, the social status is a relative concept, depending on the social target the interaction is directed at. The same person’s social preference might differ when interacting with the superior or the subordinates, for example. One possibility to account for the flexibility of social preference is offered by the query theory that suggests that valuation is constructed by posing a series of queries differing in content and sequence idiosyncratically.^53^^,^^54^^,^^55^ Our asynchronous attribute accumulation approach can be viewed as the mechanistic implementation of the query theory: the malleability of social preference in response to the change of relative social status is reflected in the order of decision attributes taken into consideration during the decision process. Alternatively, it is also conceivable that the strength of attribute weights is subject to the specific decision context. Our studies formally test these possibilities and show that the relative social status has a selective effect on the attribute latency (RST) when biasing subjects’ altruistic choices. Previous studies concerning relative social status manipulation have documented flexible social preference in different contexts.^3^^,^^4^ The selective effect of relative social status on attribute latency may stem from conflicts between situational or context-specific social norm and subject’s internal social preference.^56^^,^^57^ For instance, the inclination toward prosocial preference in reward allocation might not be suitable in the high relative social status context, where social norm encourages the possession of a larger portion of the resource. Consequently, it is plausible that the attribute latency adjustment serves as a proactive strategy to shape behavior. Via the change of RST, potentially through the aid of attention allocation,^58^^,^^59^ subjects’ real-time social preference is adjusted flexibly while simultaneously maintaining a stable intrinsic social preference, which is an important component of personality trait.

The SVO theory distinguishes between prosocial and individualistic subjects based on their attitudes toward the well-being of others.^34^^,^^60^ However, it remains agnostic about the malleability of prosociality in response to the changes in decision contexts and how SVO might influence the way attention is deployed to affect information search and subsequently subjects’ altruistic preference and RT.^24^^,^^61^ RST can be interpreted as the differential attention on different decision attribute in the evidence accumulation; our findings, therefore, suggest that the differences between prosocial and individualistic groups were due to not only the “more” decision weights assigned to others’ payoff but also “higher” RST sensitivity to the change of relative social status in the prosocial group. First, our findings are consistent with previous SVO studies showing that prosocials are indeed more altruistic than the individualists (Figures 6A and 7C). More importantly, we extend the SVO theory by revealing that prosocials also exhibit greater adaptability by adjusting the attribute latency (RST) in response to changes in relative social status, in contrast to the individualists (Figures 6B and 7D). It suggests that, in addition to being more “altruistic,” the prosocials are more sensitive to the change of social status.^56^^,^^57^ Indeed, results from study 3 further confirmed this conclusion. More importantly, we found in study 3 that subjects’ perceived social status as a function of the status level (better, equal, and worse) was more prominent in the prosocial group than the individualistic group (Figure 8A). Therefore, the higher RST sensitivities of the prosocial group might be explained by the fact that the prosocials are more sensitive to the other-regarding information than the individualists (Figures 6A, 7C, and 7G). It is possible that this higher sensitivity to other-regarding information is domain general. That is, prosocials are also more sensitive to the relative performance difference in the dot estimation task, consistent with our results (Figure 8A). Furthermore, the perceived social status and RST mediated the correlation between relative social status manipulation and subjects’ relative decision beta only in the prosocial group (Figures 8D and 8E), highlighting the importance of subjective report in revealing the psychological processes in social decision-making.

It should be noted that social preference is shaped by a variety of factors such as cognitive capacity, IQ, and psychological capacity for empathy.^62^^,^^63^^,^^64^^,^^65^^,^^66^ Although we measured both the cognitive and emotional components of subjects’ interpersonal reactivity traits (IRIs), none of the IRI subscale correlation with the RST DDM model parameters survived multiple comparison correction, indicating that a more specific task might be needed to test the relationship between these factors and subjects’ prosocial preference. Furthermore, there has been clear evidence that social preference is a dynamic construct significantly influenced by the moment-to-moment attention deployment during the decision process.^16^ Future studies could benefit from combining the eye-gaze informed attention mechanism with the assumption of asynchronous attribute latency in explaining social behavior. Finally, although the dynamical social status or rank endowed in the laboratory experiments has been reported to directly influence subjects’ economic choices, subjective well-being, and resource distribution behaviors,^1^^,^^5^^,^^6^ further studies are required to test whether these results can be generalized to the effects of social status that take much longer to establish.

In conclusion, across three studies, our experiment combines relative social status manipulation with RST DDM to demonstrate how the adaptation of social preferences to specific social contexts can be explained by changes in attribute latencies during the decision process. By extending the RST DDM from non-social to the social decision domain, we show that the adjustment of attribute start time (RST) may be a general mechanism for inducing contextually congruent behaviors without altering underlying preferences.^42^ Our results help to reconcile findings in previous literature regarding the relationship between social class and moral (altruistic) behaviors by highlighting the status reference-dependent and the dynamic nature of social status-related social preference. The expedited processing of other-regarding information, which leads to the more prosocial preference, is only evident when a prosocial subject is interacting with a partner who is higher in terms of the social status. These findings may prove useful in devising public policies and interventions that encourage faster rather than stronger social attribute processing in promoting prosocial behaviors by nudging the target audience to adopt a more humble position through methods such as awe priming.^67^

### Limitations of the study

In the current study, relative social status was manipulated using a dot number estimation task, which has been adopted in previous studies. However, it remains unclear how such a dynamic social status manipulation would correspond to the reported literature where social status was more stable and mainly determined by subjects’ social and economic status (SES). Although we also measured participants’ subjective SES in study 3 and our results showed that SES was not correlated with RST sensitivities (r = 0.129, *p* = 0.196, prosocial group; r = 0.092, *p* = 0.359, individualistic group), it is still possible that our subjects might not be fully aware of their family SES due to the fact that most of them were college students. Further studies would be required to test whether our results, obtained by eliciting relative social status in a laboratory setting, could be applied to the broad real-life scenarios. Additionally, prosocial and individualistic groups were classified based on the SVO, a measure of individuals’ preferences for others’ interests, commonly used in social decision-making research. Thus, it should be stressed that the definition of both “prosocial” and “individualistic” groups in our study is only grounded in the SVO framework. Future research exploring altruistic behavior across groups with different social orientations should consider adopting relevant scales or experimental manipulations accordingly.

## Resource availability

### Lead contact

Further information and requests for resources should be directed to and will be fulfilled by the lead contact, Yinmei Ni (niyinmei@pku.edu.cn).

### Materials availability

This study did not generate new unique reagents.

### Data and code availability

• Data for studies 1–3 have been deposited at OSF and are publicly available as of the date of publication. The DOI is listed in the key resources table.
• All original code and model weights have been uploaded to OSF and are publicly available as of the date of publication. The URL is listed in the key resources table.
• Any additional information required to reanalyze the data reported in this paper is available from the lead contact upon request.
• All data and materials that were generated for this study and all codes are posted on OSF. The URLs are listed in the key resources table.

## Acknowledgments

This work was supported by the Chinese Major Projects (2021ZD0203700) and a 10.13039/501100001809Chinese National Science Foundation grant (#32071090).

## Author contributions

J.L. and Y.N. conceived the idea and designed the experiment. Y.N. conducted the experiment and performed data analysis. J.L. and Y.N. wrote the paper. Both authors discussed the results and implications and commented on the manuscript at all stages.

## Declaration of interests

The authors declare no competing interests.

## STAR★Methods

### Key resources table

**Table:** 

REAGENT or RESOURCE	SOURCE	IDENTIFIER
**Deposited data**		

Behavioral data	This paper	OSF: https://osf.io/rwtgc/

**Software and algorithms**		

Modeling codes & codes for generating results	This paper	OSF: https://osf.io/rwtgc/
MATLAB R2018b	MathWorks	https://www.mathworks.com/products/matlab.html
R.4.1.3	The R Foundation for Statistical Computing	https://www.r-project.org/
Psychtoolbox 3.0.17	http://psychtoolbox.org/	https://github.com/Psychtoolbox-3/Psychtoolbox-3/tree/3.0.19.13
DEoptim v2.2-8	Mullen et al. (2011) https://doi.org/10.18637/jss.v040.i06	https://cran.r-project.org/web/packages/DEoptim/index.html

### Experimental model and study participant details

#### Subjects

The experiment was conducted in accordance with the protocol approved by the Ethics Committee of School of Psychological and Cognitive Sciences, Peking University. Informed written consent was obtained from each subject before each experiment.

In the original study (Study1), we recruited 65 healthy college adult students (42 females, age = 22.357 ± 2.526 years old; 23 males, age 22.826 ± 2.480). In Study 2 we recruited 114 healthy college adult students (70 females, age = 20.914 ± 2.418 years old; 44 males, age 21.273 ± 2.815) and asked them to rate how much they believe to play with a real partner (Likert scale 0–5). 9 subjects were excluded due to their disbelief of the experimental settings (Likert rating of 0), hence data of the remaining 105 subjects were used in further analyses. In Study 3 we recruited 70 healthy college adult students (34 males, age 21.909 ± 2.554; 36 females, age = 21.629 ± 2.961 years old), 2 subjects were excluded due to their disbelief of the experimental settings (Likert rating of 0), hence data of the remaining 68 subjects were used for further data analyses. Subjects received payoffs ranging from ¥55–75 (RMB) in the study according to their choices (¥50/h show up fee + choice related payoffs), and the mean payoff was ¥60.5 (RMB) across subjects.

### Method details

In all three studies, we employed a two-stage decision task that combined the task to induce relative social status (dot number estimation game) and an altruistic choice task (binary choice dictator game, DG, with subjects as the money allocators) (Figure 1A). We presented the initial findings from study 1, and our results were confirmed by study 2 where the experiment was performed in a more realistic group setting of 6–12 subjects in each group. In study 3, we additionally collected subject’s perceived social status after the dot number estimation task and confirmed the validity of our social status manipulation procedure (the dot number estimation task).

For the dot number estimation game, the total dots associated with each option were randomly drawn from a uniform distribution of [32 34], with the constraint that the number of dots between two options was not equal. Our behavioral results confirmed that subjects’ performance was around chance level (accuracy = 0.5) with reaction time window of 3s (Study 1: mean accuracy rate = 0.506, one sample t-test, $t_{64}$ = 1.698, p=0.047,cohen’sd=0.237; Study 2: mean accuracy rate = 0.504, $t_{104}$ = 1.109, p=0.155,cohen’sd=0.099; Study 3: mean accuracy rate = 0.505, $t_{67}$ = 1.109, p=0.182,cohen’sd=0.111). Unbeknownst to the subjects, we manipulated the relative performance feedback between subjects’ and their co-players such that the number of trials for each feedback type was 60 (both players were correct, BC), 60 (both were wrong, BW), 90 (subject correct and the co-player wrong) and 90 (the co-player correct & subject wrong), respectively. We further collapsed the BC and BW trials (representing equal social status) to organize subjects’ performance relative to their co-player and thus resulting in conditions of better (90 trials), equal (120 trials) and worse (90 trials) relative social status (Figure 1B).

For the binary dictator game, in each option the monetary payoffs for the subject and the co-player were randomly drawn from 1 to 20 points with the following constraints: (1) The correlation coefficients between the self and co-player payoffs in each option ranged between −0.1 and 0.1. (2) The correlation coefficients between the relative payoffs between options for the self and co-player also fell between −0.1 and 0.1 to avoid collinearity among variables. With these constraints, for each option the subject’s payoff could be larger or smaller than the co-player’s. Since the payoff amounts were randomly generated, the exact option pairs varied across subjects.

In Study 1, upon arrival, subjects were instructed to play with an anonymous co-player who finished the same task previously, and they would have the chance to pair with future subjects and might receive further payoffs allocated by the future subjects. In each trial, for the induction of relative social status, subjects were asked to choose which of the two options on the screen contained more gray dots within 3 s, after which they received performance feedback of both players for 2 s. The relative social status was determined based on subjects' relative performance in this task. Then in the dictator game (DG), subjects had to choose between two payoff allocation options for themselves and the co-players with no response time limit imposed. The inter-trial-interval is 1.5s. After the task, subjects were informed that all the payoffs in the chosen options would be summed up and converted to real money for them and the co-players to keep, and if they were selected as co-players for the future subjects, they would receive an extra amount of payoffs as the co-players. The whole task consisted of 5 sessions (60 trials each), and subjects on average took 50 min to finish the task.

In study 2, instead of recruiting one subject at a time, we recruited cohorts of 6–12 subjects. They were instructed that in each trial, they would be randomly and anonymously paired with another subject in the cohort. We anticipate that this adjustment would eliminate subjects’ impression that they may be playing with a computer and thus mimicking a more realistic social interaction environment. Also, after each dot number estimation choice, subjects were asked to complete 2–4 DG choice games with a maximum reaction time of 6 s (1.026% of trials exceeded the 6-s RT window in Study 1). The entire task was divided into 4 sessions (25 dot number estimation games per session), and it took approximately 35 min to complete the task.

The experimental design of study 3 is similar to that of study 2. In addition, subjects were required to rate their own performance in the dot estimation task on a 5-point scale (1 = very poor, 5 = very good). To confirm the validity of the relative social status manipulation of the dot estimation game, subjects also reported their perceived relative social status compared to their co-players for each type of dot performance feedback on a 7-point scale (1 = very low, 7 = very high).^6^ Finally, participants also reported their subjective socioeconomic status (SES) using a 10-rung ladder,^68^ where 1 represents the lowest tier of society (people from families at this level have the worst living conditions, the lowest education levels, the least respectable jobs, and the lowest incomes) and 10 represents the highest tier of society (people from families at this level have the best living conditions, the highest education levels, the most respectable jobs, and the highest incomes).

#### Behavioral analysis

To investigate the influence of relative social status on subjects’ choices, we calculated proportion of trials where subjects chose either the larger $M_{s}$, or the larger $M_{o}$ options respectively (Figures 2A; S6A, S6B, S7A–S7C, S8A–S8C). We also applied a linear mixed model (LMM) to examine whether social status was associated with how ${\Delta M}_{s}$ and ${\Delta M}_{o}$ affected subjects’ choices:

$$choice\;∼\;{\Delta M}_{s}\times status+{\Delta M}_{o}\times status$$

(Equation 1) $$+\left(1+{\Delta M}_{s}\times status+{\Delta M}_{o}\times status|subject\right)$$

Where status is a factor coding for the relative social status (better, equal or worse), and the choice was coded as 1 if subjects chose the larger $M_{s}$ option and 0 otherwise. Accordingly, we define $M_{s1}$ and $M_{o1}$ as the payoffs associated with the option of larger $M_{s}$, and $M_{s2}$ and $M_{o2}$ as the payoffs associated with the option of smaller $M_{s}$. ${\Delta M}_{s}\equiv$ $M_{s1}-M_{s2}$ and is always positive. Similarly, ${\Delta M}_{o}\equiv$ $M_{o1}-M_{o2}$. The results are presented in Figures 2B, 2C, S6C, S6D, S7D–S7F, S8D–S8F.

#### Computational modeling

To fit subjects’ choice and RT, we employed a multi-attribute and time-varying drift diffusion model (RST DDM). This model assumes that different attributes may enter the evidence accumulation process at different time onsets. Therefore, the decision-making process (choice and RT) can be influenced by both the attribute drift weights and their corresponding starting time. The decision evidence accumulates in discrete time (dt), and the evidence accumulation stops when the decision variable reaches the decision boundary or threshold.

At each time point t, the drift rate ($\nu_{t}$) is governed by the attributes strength ($\omega_{s}$ for ${\Delta M}_{s}$ and $\omega_{o}$ for ${\Delta M}_{o}$, respectively) as well as the drift latency parameters $\tau_{s}^{t}$ and $\tau_{o}^{t}$, which are associated with the relative start time (RST) between attributes.

(Equation 2) $$\upsilon_{t}={\tau_{s}^{t}\cdot \omega }_{s}\cdot \Delta M_{s}+\tau_{o}^{t}{\cdot \omega }_{o}\cdot \Delta M_{o}$$

Specifically, if RST>0, ${\Delta M}_{s}$ enters the decision process first; else if RST<0, $\Delta M_{o}$ enters first. Finally, if RST=0, the two attributes enter the decision process simultaneously:

(Equation 3) $$\tau_{s}^{t}=\left\{ \begin{array}{c} 1,RST\geq 0.\;\\ I\left(t\geq \left|\frac{RST}{dt}\right|\right),RST<0 \end{array}\right.,and\;\tau_{o}^{t}=\left\{ \begin{array}{c} I\left(t\geq \left|\frac{RST}{dt}\right|\right),RST>0.\;\\ 1,RST\leq 0 \end{array}\right.$$

Where I(·) is an indicator function.

The change of relative social status may influence the decision process either by modulating the attribute strength ($\omega_{s}$ and $\omega_{o}$) or by adjusting the relative start time (RST) between attributes. We therefore constructed a series of candidate models and tested their performance against our behavioral data (Table S1).

#### Model 1

The relative social status may have specific effects on the attribute strength and both attributes enter the decision process at the same time (RST=0):

(Equation 4) $$\nu_{t}=\left\{ \begin{array}{c} \omega_{s,b}\cdot \Delta M_{s}+\omega_{o,b}\cdot \Delta M_{o},Better\;\\ \omega_{s,e}\cdot \Delta M_{s}+\omega_{o,e}\cdot \Delta M_{o},Equal\;\\ \omega_{s,w}\cdot \Delta M_{s}+\omega_{o,w}\cdot \Delta M_{o},Worse\; \end{array}\right.$$

Where the $\omega_{s,b},\omega_{o,b}$, $\omega_{s,e},\omega_{o,e}$ and $\omega_{s,w},\omega_{o,w}$ are the attribute strength of $\Delta M_{s}$ and $\Delta M_{o}$ in the better, equal and worse status, respectively (Equation 1).

#### Model 2

The relative social status may only modulate the RST but not the attribute strength.

(Equation 5) $$\nu_{t}=\left\{ \begin{array}{c} \tau_{s,b}^{t}\cdot \omega_{s}\cdot \Delta M_{s}+\tau_{o,b}^{t}\cdot \omega_{o}\cdot \Delta M_{o},Better\;\\ \tau_{s,e}^{t}\cdot \omega_{s}\cdot \Delta M_{s}+{\;\tau_{o,e}^{t}\cdot \omega }_{o}\cdot \Delta M_{o},Equal\;\\ \tau_{s,w}^{t}\cdot \omega_{s}\cdot \Delta M_{s}+{\;\tau_{o,w}^{t}\cdot \omega }_{o}\cdot \Delta M_{o},Worse\; \end{array}\right.$$

Where $\tau_{s,b}^{t}$, $\tau_{o,b}^{t}$, $\tau_{s,e}^{t}$, $\tau_{o,e}^{t}$, $\tau_{s,w}^{t}$, $\tau_{o,w}^{t}$ are determined by RSTs (${RST}_{b},{RST}_{e}\;and\;{RST}_{w}$) for different status (better, equal or worse), respectively (Equation 2).

#### Model 3

We further assume that the RST is constant across relative social status but the attribute strength is not (Equation 3):

(Equation 6) $$\nu_{t}=\left\{ \begin{array}{c} \tau_{s}^{t}\cdot \omega_{s,b}\cdot \Delta M_{s}+\tau_{o}^{t}\cdot \omega_{o,b}\cdot \Delta M_{o},Better\;\\ \tau_{s}^{t}\cdot \omega_{s,e}\cdot \Delta M_{s}+{\;\tau_{o}^{t}\cdot \omega }_{o,e}\cdot \Delta M_{o},Equal\;\\ \tau_{s}^{t}\cdot \omega_{s,w}\cdot \Delta M_{s}+{\;\tau_{o}^{t}\cdot \omega }_{o,w}\cdot \Delta M_{o},Worse\; \end{array}\right.$$

#### Model 4

We assume that relative social status modulates both the attribute strength and the relative start time of the attributes (Equation 4).

(Equation 7) $$\nu_{t}=\left\{ \begin{array}{c} \tau_{s,b}^{t}\cdot \omega_{s,b}\cdot \Delta M_{s}+\tau_{o,b}^{t}\cdot \omega_{o,b}\cdot \Delta M_{o},Better\;\\ \tau_{s,e}^{t}\cdot \omega_{s,e}\cdot \Delta M_{s}+{\;\tau_{o,e}^{t}\cdot \omega }_{o,e}\cdot \Delta M_{o},Equal\;\\ \tau_{s,w}^{t}\cdot \omega_{s,w}\cdot \Delta M_{s}+{\;\tau_{o,w}^{t}\cdot \omega }_{o,w}\cdot \Delta M_{o},Worse\; \end{array}\right.$$

Where the drift latency and attribute strength parameters are the same as previously defined.

We also include the following parameters in the model: (1) Decision boundary (Threshold): the evidence threshold for the decision, which is set to be symmetric around zero. (2) nDT: the non-decision time, which accounts for the extra amount of time required for subsequent motor action not related to evidence accumulation. (3) Bias: the starting point bias for choosing larger $M_{s}$ option in the evidence accumulation process.

With the drift rate ($\nu_{t}$) specified, the evidence accumulates according to the following equation (Figures 3A and 3B):

(Equation 8) $$E_{t}=E_{t-1}+\nu_{t}\cdot dt+\varepsilon_{t}$$

Where $\varepsilon_{t}$ represents an i.i.d. Gaussian noise term from N(0,0.1). Evidence accumulation begins with the initial bias ($E_{0}=Bias$). It is then updated in discrete time steps of dt(0.01s), until *|*$E_{t}$*|* reaches the decision boundary and the response time (RT) is computed as t·dt+nDT.

For each candidate model and each subject, we estimated the best-fitting parameters using the differential evolution algorithm (an R Package developed by Mullen and colleagues^43^) with 150 iterations. In each iteration, we simulated 3,000 choices and RTs for each trial. Trials with actual RT longer than 10s and beyond three standard deviations were excluded from model-fitting, and this exclusion criteria resulted in a total of 52 trials discarded for all the subjects (less than 0.3% of all trials in study1, and no trial was discarded in studies 2 & 3). The likelihood of the observed choices and RTs were generated according to the distribution generated by the 3000 simulated choices. For each subsequent iteration, the parameter populations evolved toward maximizing the likelihood of the observed data (subjects’ choices and RTs).^43^ We repeated the above process in each model 100 times to obtain the best parameter estimations.

Bayesian model selection was then used for model comparison^45^ (Figures S2 and S9) and the candidate model performance was summarized in Table S2.

#### Model simulated decision betas across RTs

We simulated model predicted decision betas of $\Delta M_{s}$ and $\Delta M_{o}$ as functions of RT. We simulated each trial 100 times for all subjects’ option sets with the mean parameters of Model 2. To more intuitively show the effect of attribute weight and latency on choice, the initial bias was set to 0. Then, we ran logistic regressions ($Choice\;∼\Delta M_{s}+\Delta M_{o}$) in 100ms moving time windows in steps of 10ms. This result is shown in Figures 3C–3E.

#### Model parameter recovery

We ran parameter recovery for the best model by simulating choices and RTs based on the estimated parameters for each subject and each trial. Then, we separately fitted the simulated behaviors using the best model and recovered the model parameters for each subject. We then calculated the correlation between the recovered parameters and true parameters originally estimated from fitting subjects’ actual behavior. This result is shown in Figures S3 and S11.

#### Model cross-validation

To conduct the cross-validation analysis for the winning RST model, we randomly split subjects’ choice and RT data into two-halves for each relative social status condition in each subject.^12^^,^^31^ Next, we used the parameters estimated from half of the data to simulate subjects’ choices and RTs for the option sets of the other half data 100 times and computed the simulated choice accuracy against subjects’ actual choices and the RT correlation with subjects’ true RTs in the other half data. The results are shown separately for better, equal, and worse conditions in Figures 4 and 10.

Next, we tested how the best model could capture subjects’ behavior patterns. We averaged the simulated choices and RTs for the 100 stimulations of each trial and compared the model predicted choices in each relative social status condition (Figures 2A, S6A, S6B, S7A–S7C, S8A–S8C, red error bars). We also retrieved the decision regression coefficients of $\Delta M_{s}$ and $\Delta M_{o}$ and plotted both the simulated and actual regression coefficients together for comparison (Figures 2B, 2C, S6C, S6D, S7D–S7F, S8D–S8F, red error bars). The detailed description of this regression analysis is in the regression analysis section (Equation 1). Finally, we plotted the simulated RTs across relative social status (Figures 2D; S6E; S7G, S7H, S8G, and S8H, red error bars) along with the actual RTs.

#### Mediation analysis

We conducted the simple mediation analyses using the mediation function in the R package "mediation"^69^ to explore the mediating effect of RST (mediator) on the relationship between relative social status (the independent variable) and subjects’ performance variables (the dependent variable). Separate mediation models were developed for each of the three dependent variables: the relative decision beta between $\Delta M_{s}$ and $\Delta M_{o}$ attributes, and the proportion of trials where subjects chose the option with a larger $M_{s}$ or the option with a larger $M_{o}$. In all the mediation models tested, we controlled the effect of attribute strength ($\omega_{s}$ and $\omega_{o}$). Bootstrapping with 1000 iterations was used to estimate the model confidence intervals, and the results are presented in Figures 5 and S13. For the chain mediation analyses (Figures 8D and 8E), “sem” function in R package “lavaan” was used.^70^ In this analysis, both the perceived relative social status (M1) and RST (M2) were treated as mediators, and the independent and dependent variables were the same as in previous simple mediation model (Figures 8D, 8E, and S16).

#### Questionnaires

Subjects’ social preference and interpersonal reactivity traits were measured with the social value orientation^34^ (SVO) and interpersonal reactivity index (IRI) questionnaires.^71^^,^^72^ SVO comprises six items that involve deciding the monetary allocation between self and others. In each item, subjects are required to select their preferred allocation (such as 100–50 or 98–54) from nine available options. The SVO angle is then calculated based on the average amount of money form the chosen options for oneself ($\bar{A_{S}}$) and for others ($\bar{A_{o}}$).

(Equation 9) $${\text{SVO}}^{0}=\arctan\;\left(\frac{\bar{A_{o}}-50}{\bar{A_{S}}-50}\right)$$

Based on the SVO angle, subjects can be classified into four types: altruists (${\text{SVO}}^{0}>57.15^{0}$), prosocials (${\text{SVO}}^{0}∊{[22.45}^{0}$, $57.15^{0}$]), individualists (${\text{SVO}}^{0}∊{[-12.04}^{0}$, $22.45^{0}$], and competitive types (${\text{SVO}}^{0}<{-12.04}^{0}$).^34^^,^^73^ Studies utilizing the SVO angle generally combine altruists and prosocials into a single “prosocial” category (${\text{SVO}}^{0}>22.45^{0}$), and group individualists and competitors into the individualistic or “proself” category (${\text{SVO}}^{0}<22.45^{0}$), as the proportions of altruists and competitors were relatively small among subjects.^47^^,^^74^^,^^75^^,^^76^ In our current study, we adopted this two-type SVO classification (Figures 6, 7C, 7D, and S6–S8).

The IRI measures different aspects of interpersonal reactivity and includes 4 subscales: perspective-taking (PT), empathic concern (EC), fantasy (FS) and personal distress (PD).

### Quantification and statistical analysis

The computational modeling of RST DDM was conducted using DEoptim (v2.2-8) package in R (version 4.1.3).^43^ Mixed-effect regression analyses were conducted with “fitglme” function in MATLAB (R2018b). All correlation analyses were conducted using robust correlation with “pbcor” function in R package of “WRS2”.^77^ Mediation analyses was conducted with the “mediation”^69^ and “sem” function in R packages of “mediation” and “lavaan”.^70^ Non-parametric analyses of aligned rank transformation (ART) test and Friedman test were used when the normality assumption was not met for the otherwise one-way or two-way ANOVA test,^48^^,^^49^ and the Bonferroni correction was used when the need for multiple comparison correction arose.
