# Supplementary material for: Relative social status alters the synchrony of attribute integration in altruistic decisions
Source: iScience. 2025 Jan 27;28(3):111911. doi: 10.1016/j.isci.2025.111911 (PMC11876899; doi:10.1016/j.isci.2025.111911)
Supplement: Document S1. Figures S1–S16, Tables S1, and S2 [file mmc1.pdf]

iScience, Volume *28*

Supplemental information

Relative social status alters the synchrony of attribute integration in altruistic decisions

Yinmei Ni and Jian Li

Table S1. Specification and performance of candidate models, related to STAR Methods of Computational modeling. In the RST DDMs, ω_s and ω_o are the drift weights for ΔM_s and ΔM_o attributes, respectively; RST is the relative start time between ΔM_s and ΔM_o, with positive value representing earlier entry of ΔM_s, and negative value representing earlier entry of ΔM_o into the decision process. Bias: the initial starting bias for choosing option with larger M_s in the evidence accumulation process for Model 1-4; for Model 5, we added two bias parameters associated with choosing larger M_s and larger M_o options, respectively. nDT: the non-decision time, which accounts for the extra amount of time required for subsequent motor action not related to evidence accumulation. Threshold: the evidence threshold or decision boundary for the decision. BIC: Bayesian information criteria.

	ω_s	ω_o	RST	bias	nDT	threshold	Total Parameters	BIC (Study1)	BIC (Study2)	BIC (Study3)
Model1	3	3	0	1	1	1	9	207223	337153	215779
Model2 (RST Model)	1	1	3	1	1	1	8	207030	333708	215500
Model3	3	3	1	1	1	1	10	207375	337513	216244
Model4	3	3	3	1	1	1	12	208146	338731	217218
Model5	1	1	3	2	1	1	9	208782	339376	217106

Table S2. Estimated model parameters for the winning model (Model 2, the RST model), related to STAR Methods of Computational modeling and Figure 4A&B, Figure 6 and Figure 7. ω_s and ω_o are the drift weights for ΔM_s and ΔM_o attributes respectively; RST is the relative start time between ΔM_s and ΔM_o, with positive value representing earlier entry of ΔM_s, and negative value representing earlier entry of ΔM_o into the decision process. Bias: the initial starting bias for choosing larger M_s option in the evidence accumulation process. nDT: the non-decision time, which accounts for the extra amount of time required for subsequent motor action not related to evidence accumulation. Threshold: the evidence threshold or decision boundary for the decision.

Parameter	Study	All mean±s.d.	Prosocials mean±s.d.	Proselfs mean±s.d.
ω_s	1	0.314±0.025	0.230±0.020	0.349±0.027
	2	0.284±0.012	0.260±0.015	0.226±0.014
	3	0.351±0.028	0.227±0.020	0.473±0.028
ω_o	1	0.120±0.023	0.103±0.011	0.078±0.021
	2	0.037±0.018	0.076±0.020	0.091±0.014
	3	0.079±0.020	0.091±0.017	0.066±0.028
RST_b	1	0.618±0.120	0.793±0.202	0.564±0.097
	2	0.496±0.063	0.510±0.109	0.825±0.115
	3	0.560±0.097	0.813±0.165	0.303±0.060
RST_e	1	0.179±0.121	0.227±0.199	0.255±0.078
	2	0.294±0.059	0.080±0.095	0.361±0.098
	3	0.275±0.081	0.376±0.145	0.150±0.049
RST_w	1	-0.343±0.155	-0.613±0.232	-0.185±0.110
	2	-0.195±0.093	-0.553±0.148	-0.486±0.131
	3	-0.168±0.105	-0.467±0.179	0.116±0.067
**bias**	1	0.530±0.036	0.484±0.050	0.417±0.028
	2	0.422±0.024	0.389±0.033	0.340±0.025
	3	0.411±0.028	0.340±0.036	0.494±0.029
**threshold**	1	1.590±0.053	1.749±0.071	1.380±0.048
	2	1.397±0.034	1.469±0.049	1.488±0.044
	3	1.382±0.048	1.483±0.062	1.271±0.048
**nDT**	1	0.573±0.017	0.573±0.026	0.515±0.019
	2	0.526±0.013	0.529±0.020	0.533±0.024
	3	0.514±0.019	0.535±0.034	0.497±0.012

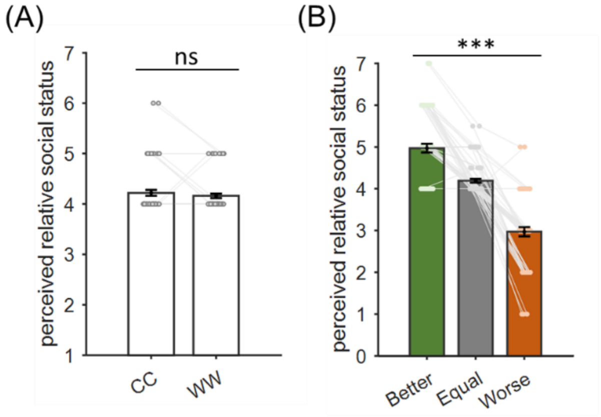

Figure S1. Perceived relative social status in Study 3, related to Figure 8. (A), Subjects' perceived relative social status showed no difference (ranksum test, $p = 0.605$) when the subjects and their co-players were both correct (BC) and wrong (BW) in the dot estimation task. (B), There was a significant main effect of subjects' perceived relative social status across better, equal, and worse conditions ($Friedman\ \ \chi^2 = \ \ 100.92,\ df = 2,\ p\ \ <\ \ 0.001$). ***p<0.001. Error bars represent s.e.m across subjects.

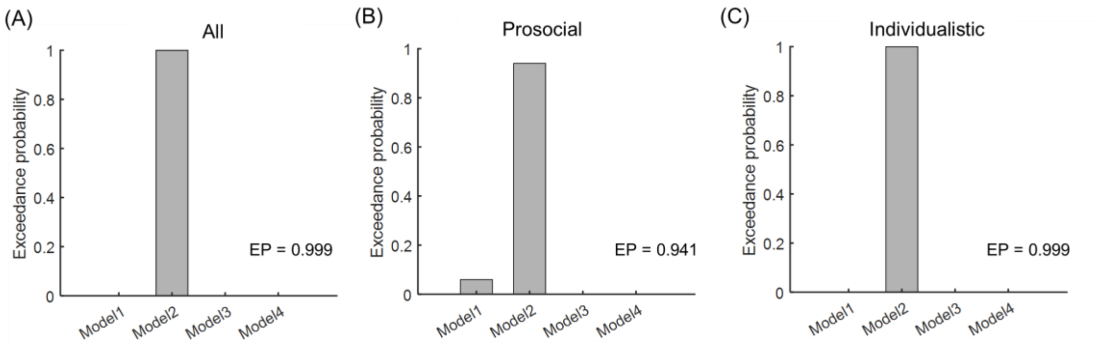

Figure S2. Bayesian model selection results in Study 1, related to STAR Methods of Computational modeling. Across all the subjects (A) or within the prosocial group (B) or individualistic group (C), model 2 (RST model) outperformed other models with the highest exceedance probability.

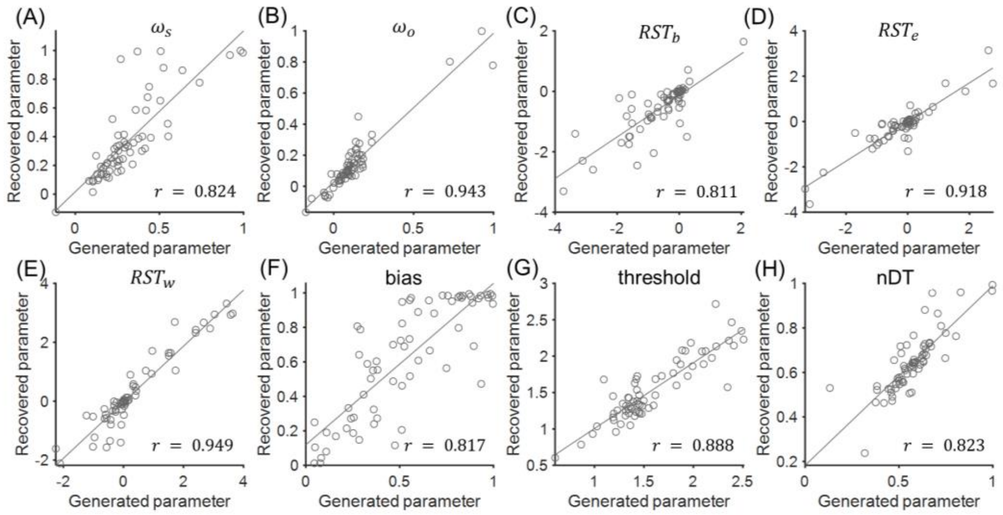

Figure S3. Parameter recovery analysis for the winning RST model in Study 1, related to STAR Methods of Model parameter recovery. Correlations between actual parameter and the model recovered parameters.

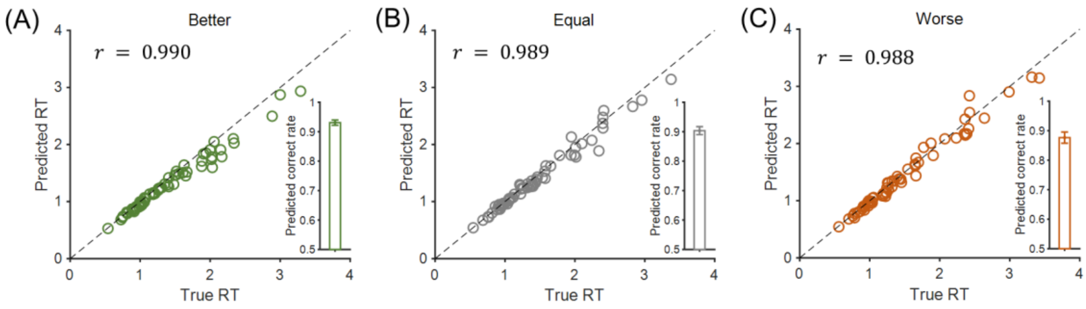

Figure S4. Cross-validation results in Study 1, related to STAR Methods of Model cross-validation. The correlation between predicted and participants' actual RTs (empty circle) and choices (bar) across subjects in the better, equal and worse conditions.

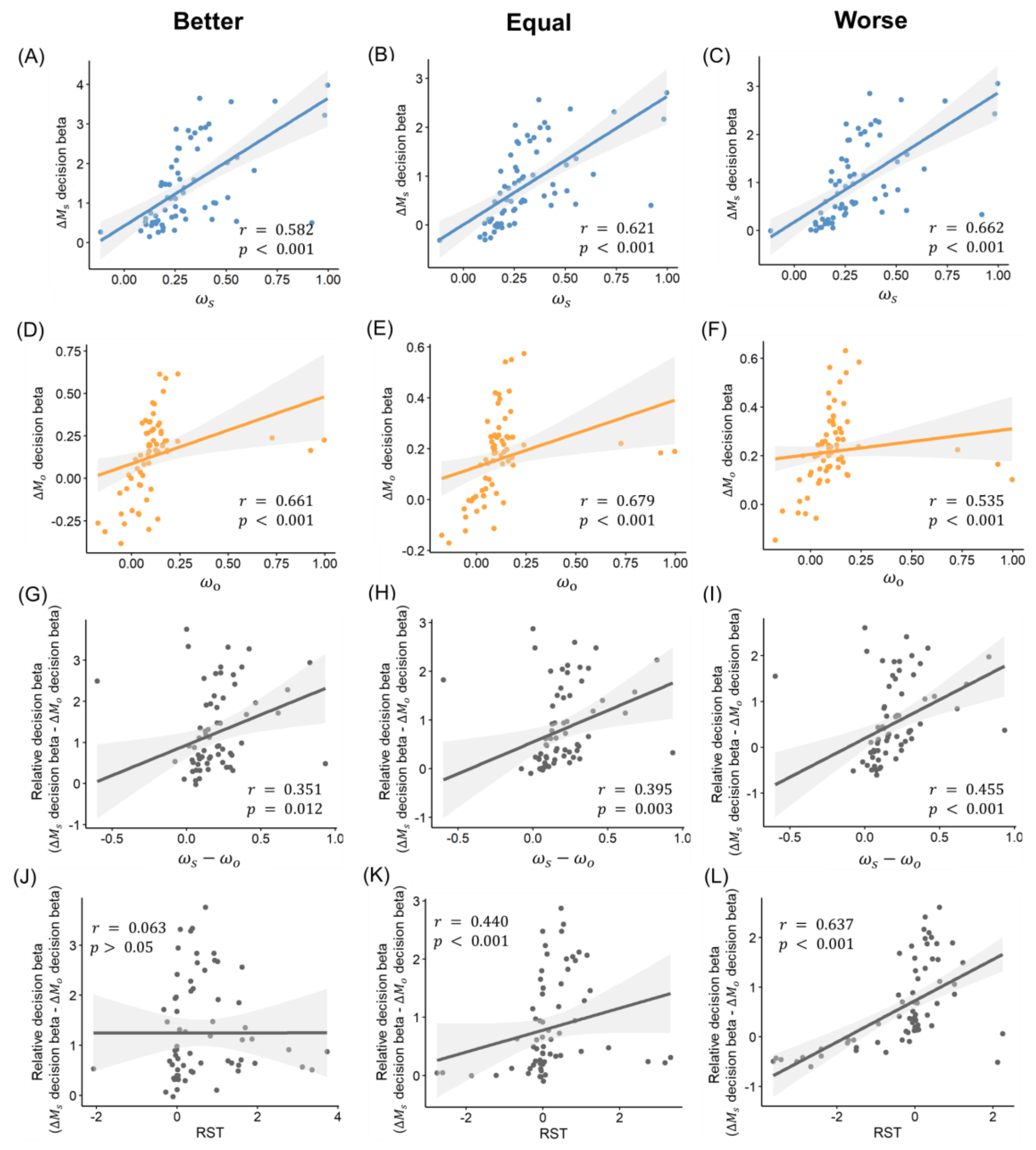

Figure S5. The correlation between model parameters and decision betas in Study 1, related to Figure 4C-F. (A-C), Robust correlation of drift weight and decision beta for ΔM_s in better (A), equal (B) and worse (C) conditions. (D-F), Robust correlation of drift weight and decision beta for ΔM_o in better (D), equal (E) and worse (F) conditions. (G-I), Robust correlation of relative drift weight and relative decision beta for $\Delta M_s - \Delta M_o$ in better (G), equal (H) and worse (I) conditions. (J-L), Robust correlation of RST and relative decision beta ($\Delta M_s - \Delta M_o$) in better (J), equal (K) and worse (L) conditions.

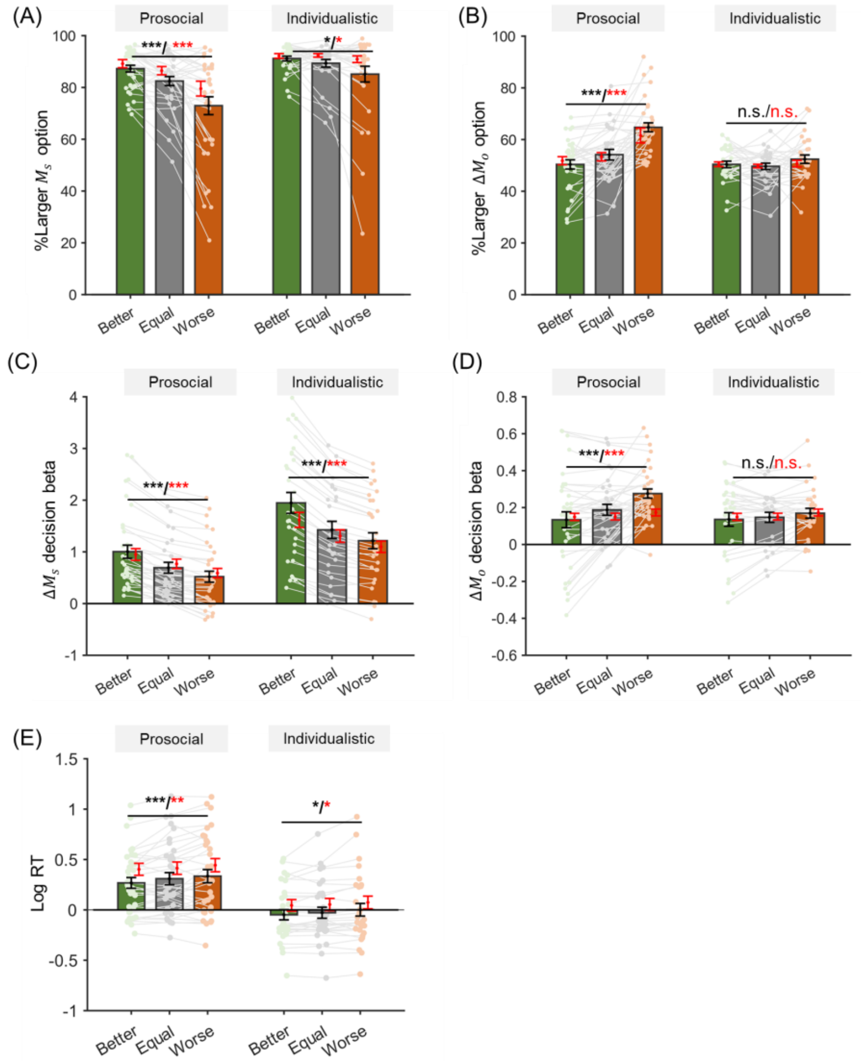

Figure S6. Behavioral differences between prosocial and individualistic groups in Study 1, related to Figure 2. (A) Proportion of larger self payoff option selection across relative social status for the prosocial and individualistic groups. (B) Proportion of larger co-player payoff option selection across relative social status for the prosocial and individualistic groups. (C) Decision betas of ΔM_s across relative social status for the prosocial and individualistic groups. (D) Decision betas of ΔM_o across relative social status for the prosocial and individualistic groups. (E) Mean log RT across relative social status for the prosocial and individualistic groups. Asterisks across relative social status denote significant main effect of one-way ANOVA. Asterisks across prosocial and individualistic groups denote significant interaction effect of two-way ANOVA (relative social status×SVO group). Error bars represent s.e.m across subjects and red error bars represent model predictions from the cross-validation results (see methods). *** $p < 0.001$, ** $p < 0.01$ and * $p < 0.05$.

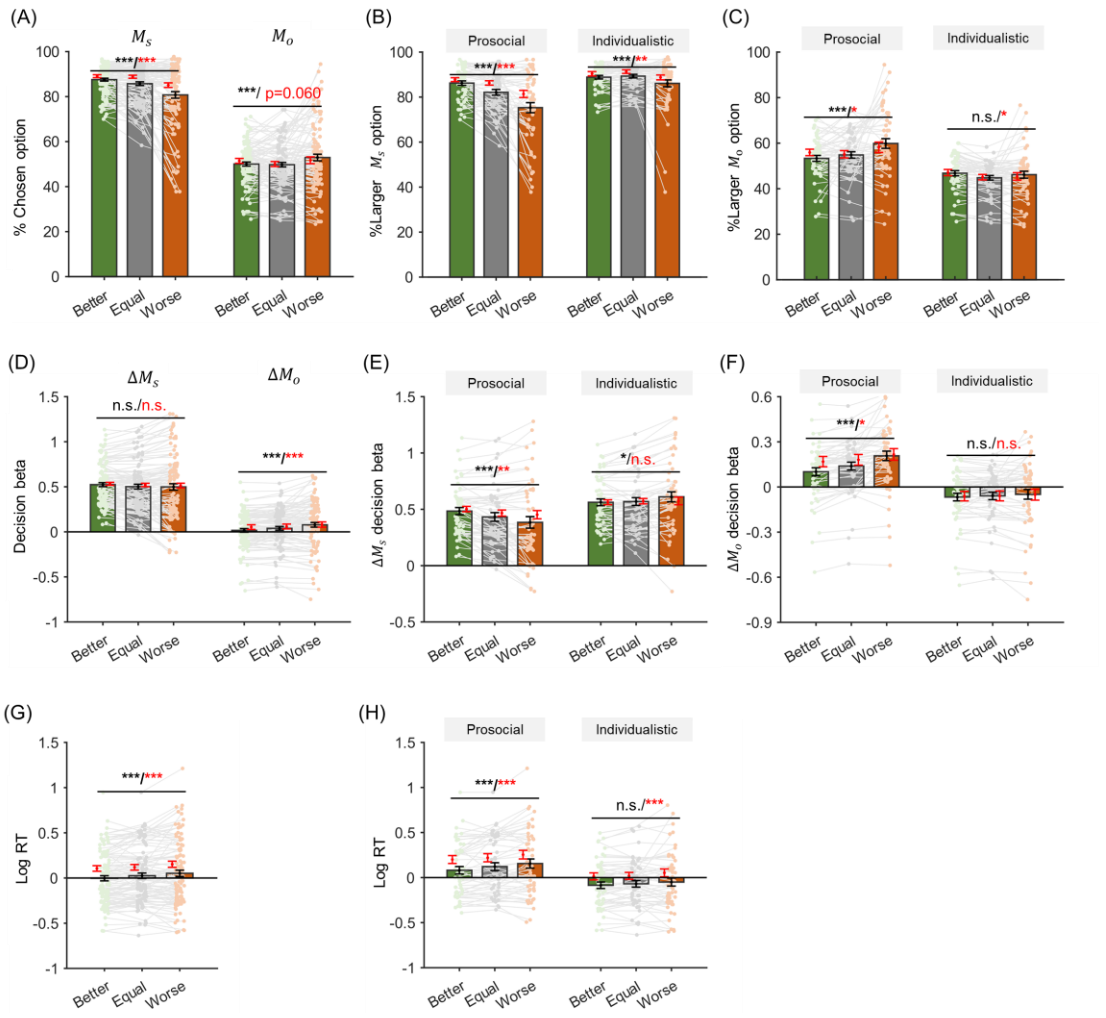

Figure S7. Behavioral results for Study 2, related to Figure 2. (A), Proportions of chosen larger self payoff (M_s) and other payoff (M_o) option. (B), Proportions of chosen larger self payoff option for the prosocial and individualistic groups. (C), Proportion of chosen larger other payoff option for the prosocial and individualistic groups. (D), Decision betas of ΔM_s and ΔM_o. (E) Decision betas of ΔM_s for the prosocial and individualistic groups. (F), Decision betas of ΔM_o for the prosocial and individualistic groups. (G), Mean log RT. (H), Mean log RT for the prosocial and individualistic groups. Asterisks across relative social status denote significant main effect of one-way ANOVA. Asterisks across prosocial and individualistic groups denote significant interaction effect of two-way ANOVA (relative social status×SVO group). Error bars represent s.e.m across subjects and red error bars represent model predictions from the cross-validation results (see methods). *** $p < 0.001$, ** $p < 0.01$ and * $p < 0.05$.

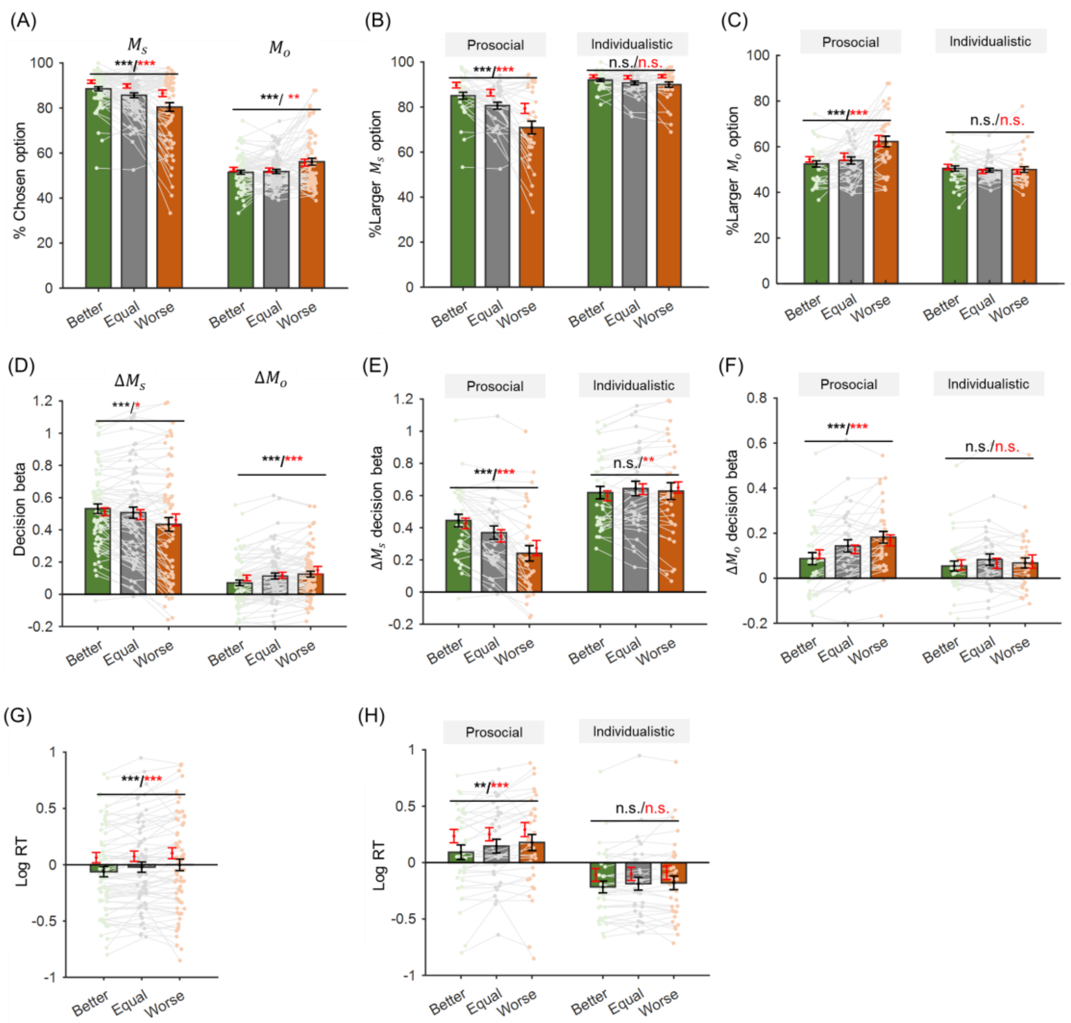

Figure S8. Behavioral results for Study 3, related to Figure 2. (A), Proportions of chosen larger self payoff (M_s) and other payoff (M_o) option. (B), Proportions of chosen larger self payoff option for the prosocial and individualistic groups. (C), Proportion of chosen larger other payoff option for the prosocial and individualistic groups. (D), Decision betas of ΔM_s and ΔM_o. (E) Decision betas of ΔM_s for the prosocial and individualistic groups. (F), Decision betas of ΔM_o for the prosocial and individualistic groups. (G), Mean log RT. (H), Mean log RT for the prosocial and individualistic groups. Asterisks across relative social status denote significant main effect of one-way ANOVA. Asterisks across prosocial and individualistic groups denote significant interaction effect of two-way ANOVA (relative social status×SVO group). Error bars represent s.e.m across subjects and red error bars represent model predictions from the cross-validation results (see methods). *** $p < 0.001$, ** $p < 0.01$ and * $p < 0.05$.

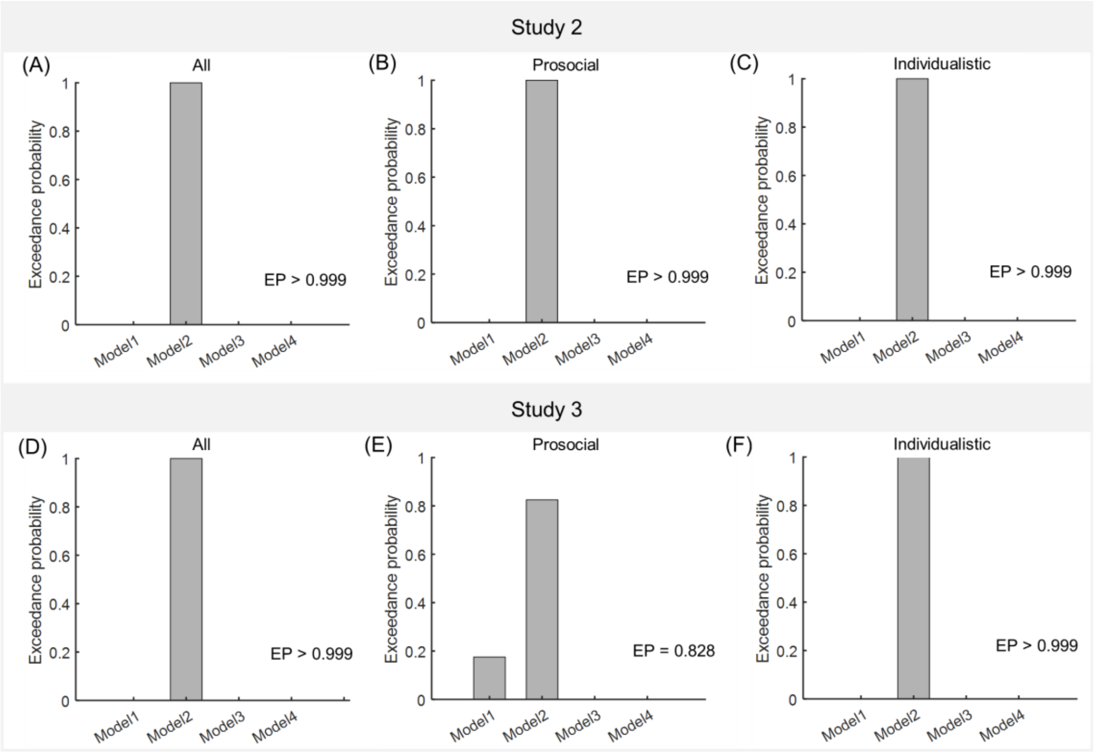

Figure S9. Bayesian model selection results for the Study 2 and 3, related to STAR Methods of Computational modeling. Across all the subjects (A) or within the prosocial group (B) or individualistic group (C), Model 2 (RST model) outperformed other models with the highest exceedance probability.

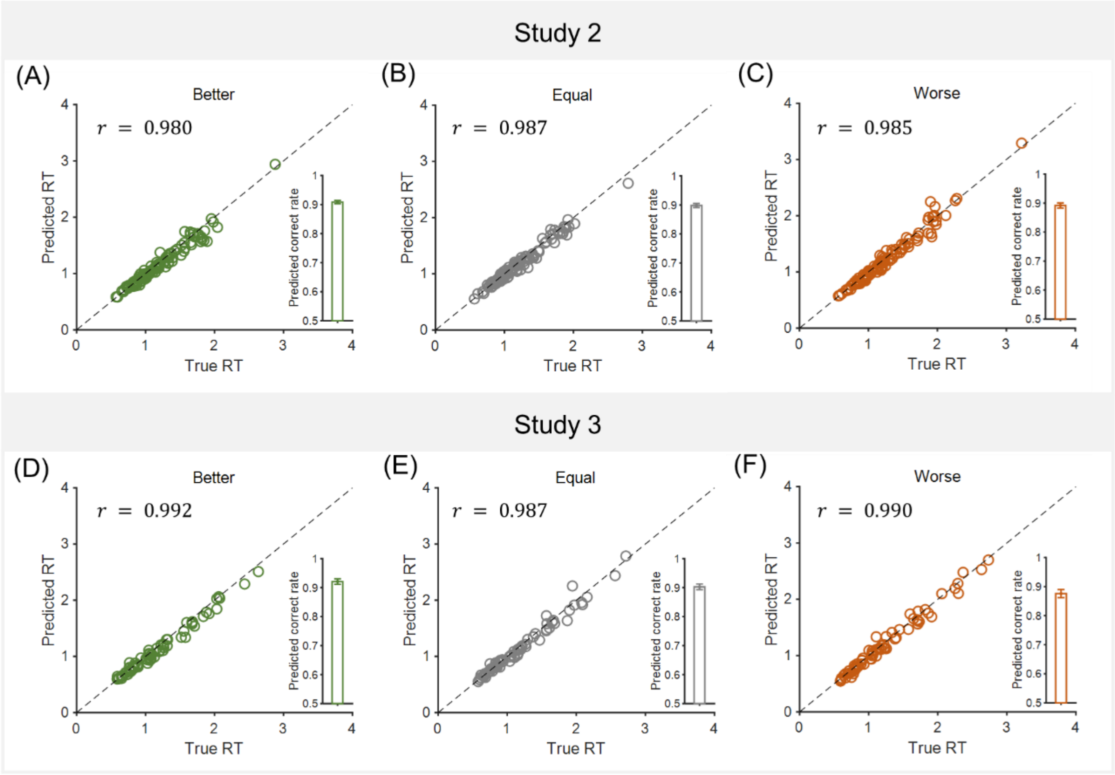

Figure S10. Cross-validation results for replication Study 2 and 3, related to STAR Methods of Model cross-validation. The correlation between predicted and participants' actual RTs (empty circle) and choices (bar) across subjects in the better, equal and worse conditions.

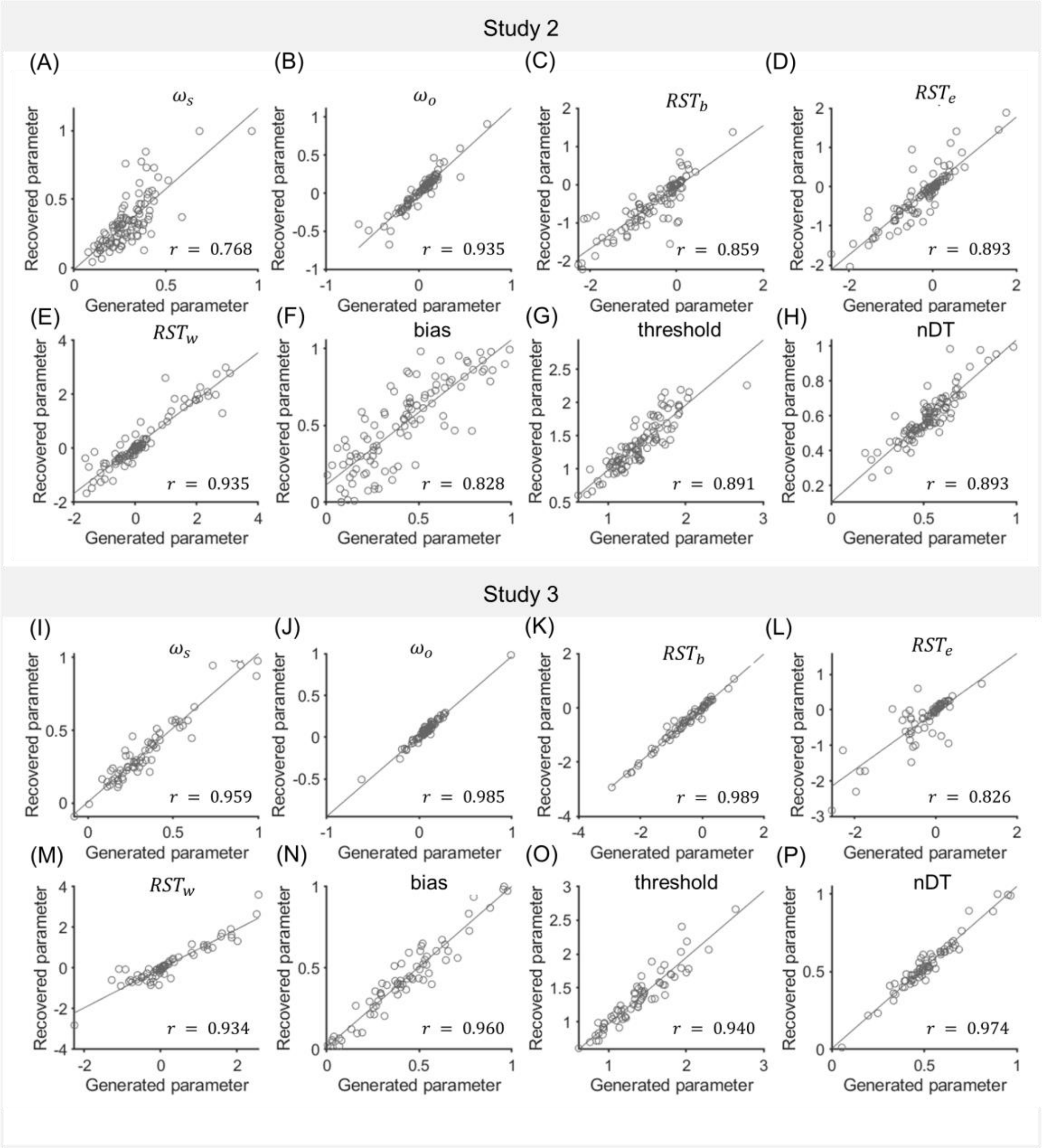

Figure S11. Parameter recovery analysis of the RST model for Study 2 and 3, related to STAR Methods of Model parameter recovery. Correlations between actual parameter and the model recovered parameters.

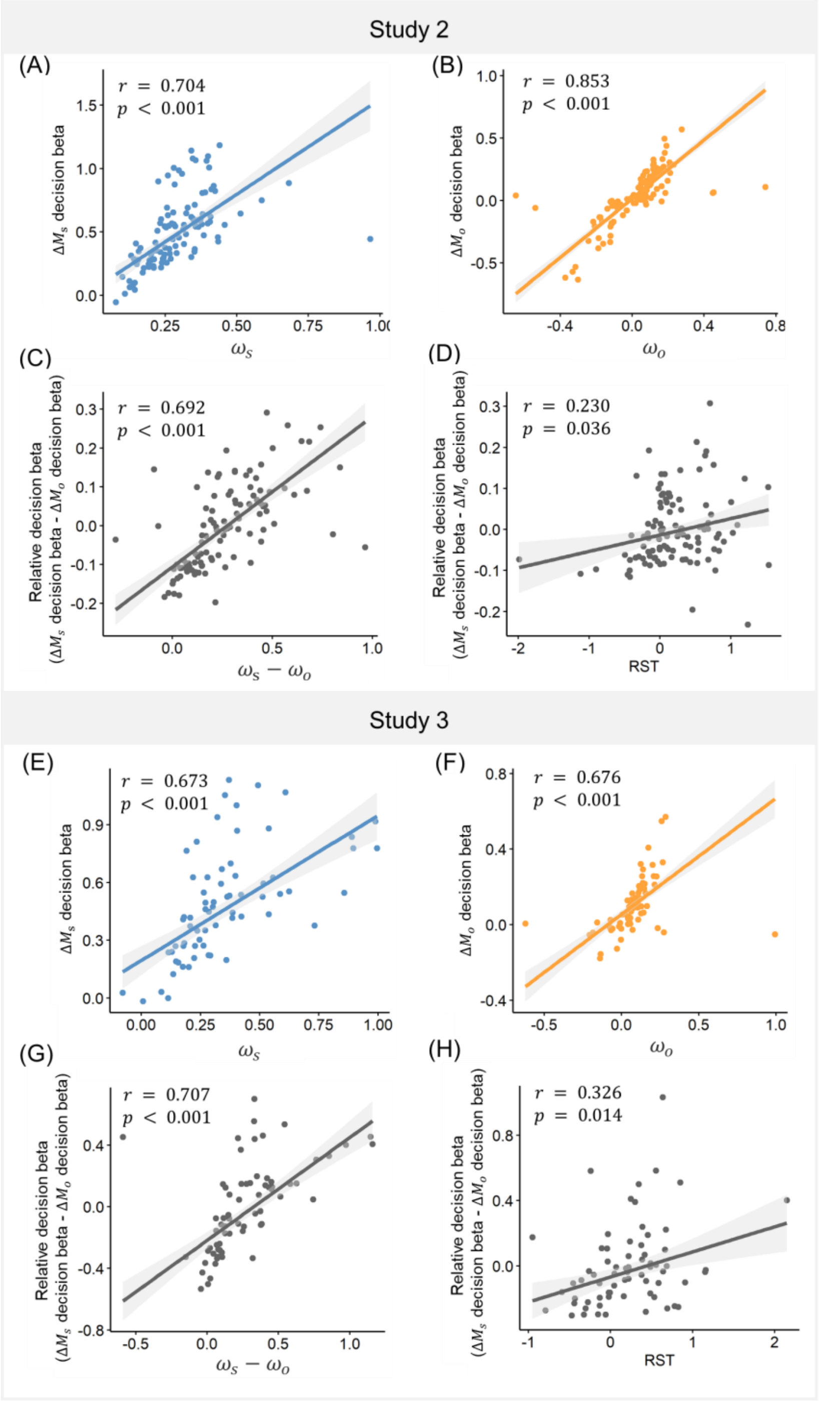

Figure S12. Correlations between model parameters and decision betas for Study 2 and 3, related to Figure 4C-F. Robust correlation between drift weights and decision betas for ΔM_s (A) and ΔM_o (B) after controlling for the effect of RST. (C), Correlation between relative drift weights (ΔM_s - ΔM_o) and relative decision betas (ΔM_s - ΔM_o), controlling for the effect of RST. (D), Correlation between RSTs and relative decision betas, controlling for the effect of relative drift weight.

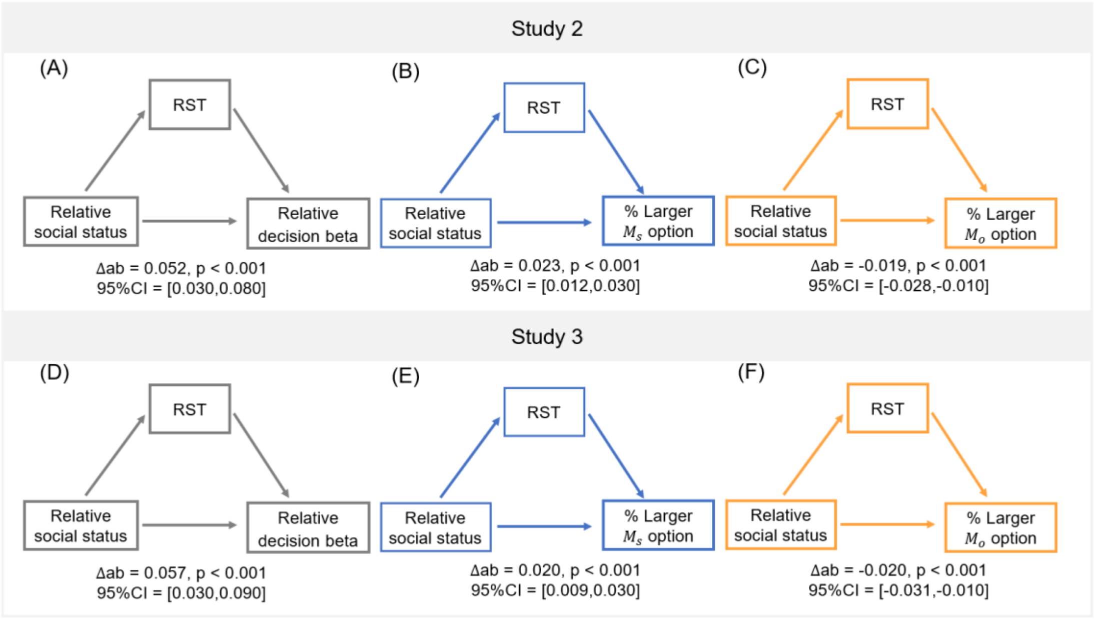

Figure S13. Mediation analysis for Study 2 and 3, related to Figure 5. (A), RST mediated the effect of relative social status on subjects' relative decision beta (ΔM_s decision beta - ΔM_o decision beta). (B), RST partially mediated the effect of relative social status on chosen larger self payoff proportions. (C), RST mediated the effect of relative social status on chosen larger co-player payoff proportions.

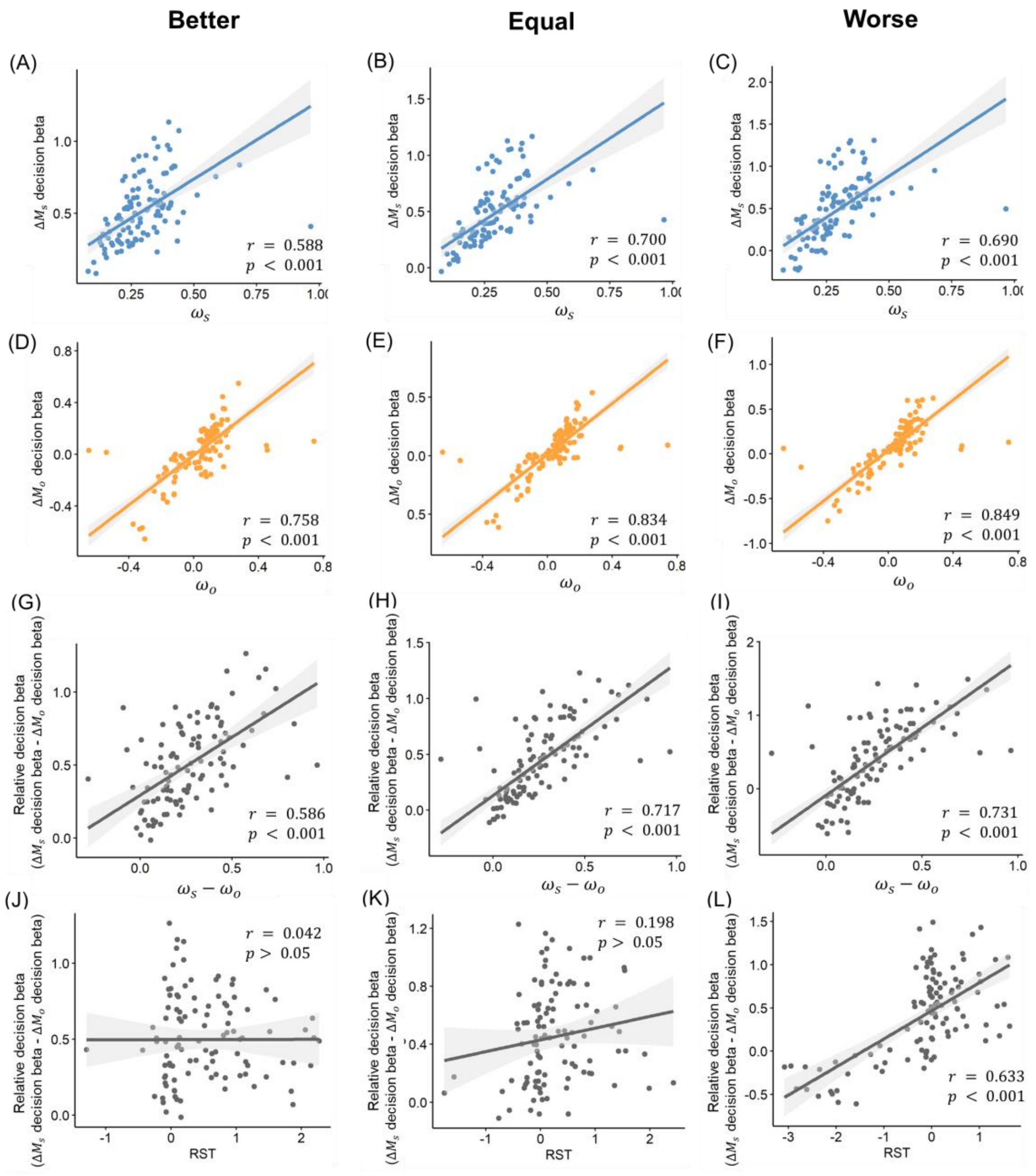

Figure S14. Model parameters correlation with decision regression coefficients for Study 2, related to Figure 4C-F. (A-C), Robust correlation of drift weight and decision beta for ΔM_s in better (A), equal (B) and worse (C) conditions. (D-F), Robust correlation of drift weight and decision beta for ΔM_o in better (D), equal (E) and worse (F) conditions. (G-I), Robust correlation of relative drift weight and relative decision beta for $\Delta M_s - \Delta M_o$ in better (G), equal (H) and worse (I) conditions. (J-L), Robust correlation of RST and relative decision beta ($\Delta M_s - \Delta M_o$) in better (J), equal (K) and worse (L) conditions.

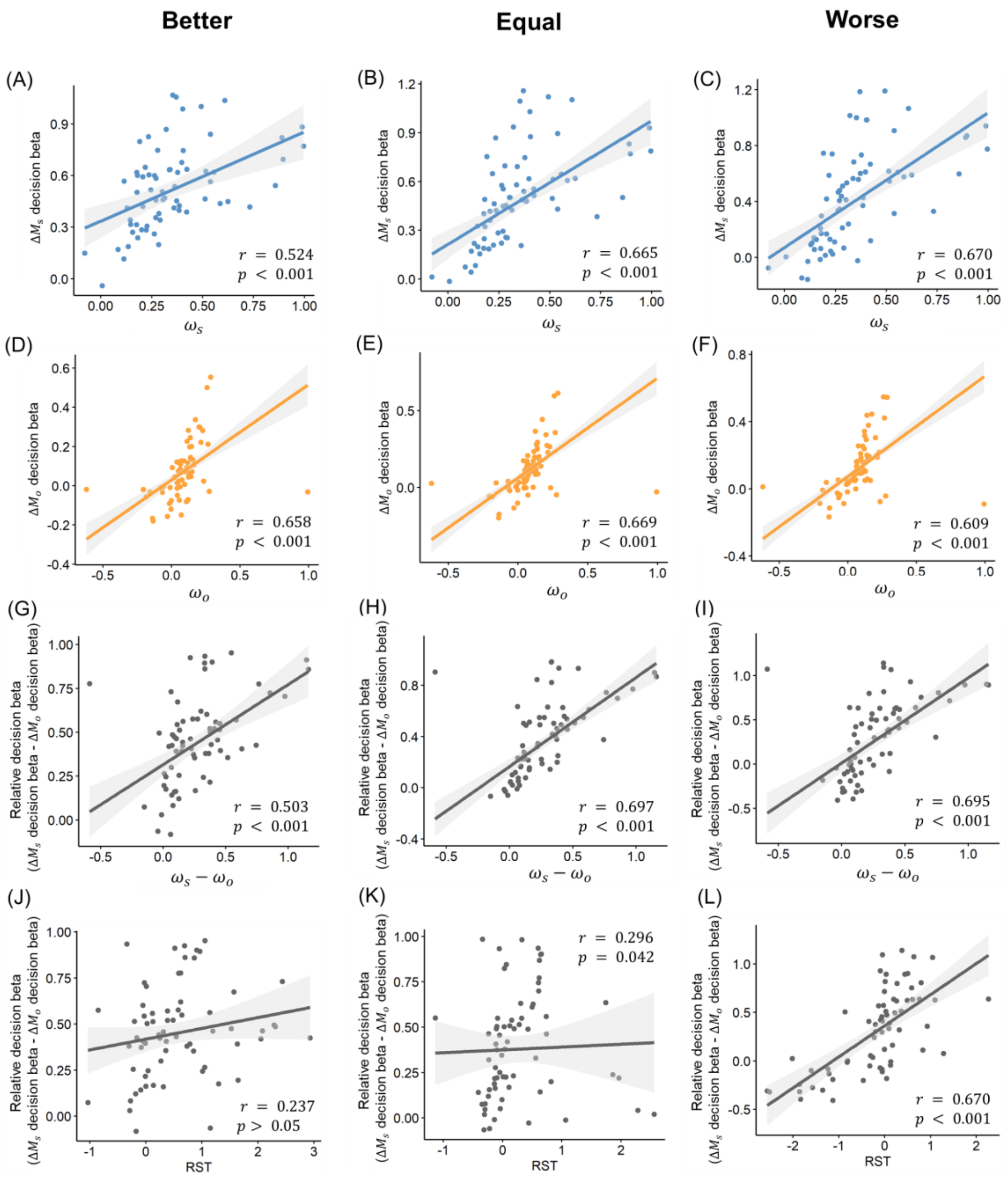

Figure S15. Model parameters correlation with decision regression coefficients for Study 3, related to Figure 4C-F. (A-C), Robust correlation of drift weight and decision beta for ΔM_s in better (A), equal (B) and worse (C) conditions. (D-F), Robust correlation of drift weight and decision beta for ΔM_o in better (D), equal (E) and worse (F) conditions. (G-I), Robust correlation of relative drift weight and relative decision beta for $\Delta M_s - \Delta M_o$ in better (G), equal (H) and worse (I) conditions. (J-L), Robust correlation of RST and relative decision beta ($\Delta M_s - \Delta M_o$) in better (J), equal (K) and worse (L) conditions.

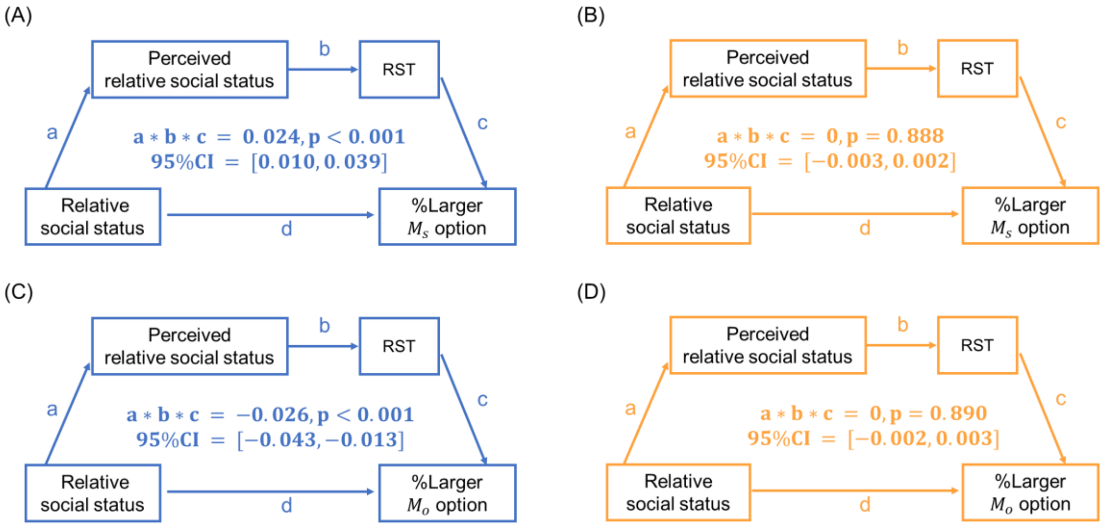

Figure S16. The chain mediation analysis for Study 3, related to Figure 8D&E. The perceived relative social status and RST significantly mediated the relative social status's effect on chosen larger M_s proportions in the prosocial group (A), but not in the individualistic group (B). Similarly, the perceived relative social status and RST significantly mediated the relative social status's effect on chosen larger M_o proportions in the prosocial group (C), but not in the individualistic group (D).
